# Stress-related helplessness is independently associated with reward-driven eating in adults with BMI-defined normal weight and obesity

**DOI:** 10.3389/fpsyg.2026.1820806

**Published:** 2026-10-14

**Authors:** Imke Schamarek, Peter Kovacs, Michael Stumvoll, Matthias Blüher, Anke Tönjes, Kerstin Rohde-Zimmermann

**Affiliations:** 1 Medical Department III–Division of Endocrinology, Nephrology, Rheumatology, University of Leipzig Medical Center, Leipzig, Germany; 2 German Center for Diabetes Research (DZD), Neuherberg, Germany; 3 Helmholtz Institute for Metabolic, Obesity and Vascular Research (HI-MAG), Helmholtz Center Munich at the Leipzig University and University of Leipzig Medical Center, Leipzig, Germany; 4 LeiCeM - Leipzig Center of Metabolism, University of Leipzig, Leipzig, Germany

**Keywords:** food craving, hedonic hunger, obesity, perceived stress, reward-driven eating, uncontrolled eating

## Abstract

**Background:**

Accumulating evidence indicates that stress is associated with reward-driven eating, yet the stress dimensions underlying this relationship remain insufficiently understood. Inconsistent findings may partly reflect conceptualizing stress as a unitary construct and insufficient consideration of weight status. This study examined the independent associations of perceived stress dimensions (Helplessness, Self-Efficacy) with hedonic hunger, trait food craving, and uncontrolled eating in adults with BMI-defined normal weight and obesity.

**Methods:**

Ninety adults were enrolled, 47 with normal weight, 18 with overweight, and 25 with obesity (BMI ≥ 30 kg/m^2^). Primary inferential analyses included participants with normal weight and obesity (*N* = 72); participants with overweight were excluded. Participants completed questionnaires assessing perceived stress (Perceived Stress Scale, PSS), hedonic hunger (Power of Food Scale), trait food craving (Food Craving Questionnaire-Trait reduced), and uncontrolled eating (Three-Factor Eating Questionnaire). PSS Helplessness and Self-Efficacy subscales were derived. Associations were examined using Spearman correlations and multiple linear regression adjusted for age, sex, BMI, and current hunger. Helplessness and Self-Efficacy were entered simultaneously to estimate independent associations with reward-driven eating outcomes. PROCESS moderation analyses tested whether BMI or BMI-defined weight status influenced the association.

**Results:**

In the primary analysis sample, PSS Total was not independently associated with any reward-driven eating outcome after correction for multiple testing. In contrast, Helplessness was independently associated with greater hedonic hunger, food craving, and uncontrolled eating after FDR correction, whereas Self-Efficacy was not independently associated with any outcome after accounting for Helplessness. In subgroup analyses, associations were descriptively more consistent among participants with obesity than those with normal weight. However, no interaction effect remained significant after correction for multiple testing.

**Conclusion:**

These findings highlight Helplessness, reflecting, perceived uncontrollability, as the perceived stress dimension most consistently associated with reward-driven eating and underscore the importance of distinguishing between stress dimensions rather than relying solely on overall perceived stress. There was no robust evidence in the present study that BMI or BMI-defined weight status modified these associations. Perceived uncontrollability may represent a potential target for interventions aimed at reducing maladaptive reward-driven eating behavior.

## 1 Introduction

The modern food environment, characterized by ubiquitous access to highly palatable, energy-dense foods, may amplify the impact of food reward on eating behavior (Espel-Huynh et al., 2018; de Oliveira et al., 2022). Reward-driven eating can occur in the absence of immediate homeostatic requirements and is primarily motivated by the pursuit of pleasure or the alleviation of negative affect states (Rossi and Stuber, 2018; Tomiyama et al., 2011; Tuluhong and Han, 2023; Leehr et al., 2015). Hedonic hunger, trait food craving, and uncontrolled eating constitute key, interrelated constructs reflecting reward-driven eating (Crane et al., 2023). Hedonic hunger describes the motivation to consume palatable foods for pleasure, triggered by environmental cues, despite satiety (Lowe and Butryn, 2007). Trait food craving denotes a stable predisposition to experience intense desires for specific, typically highly palatable foods perceived as rewarding (Weingarten and Elston, 1990). Trait uncontrolled eating emerges when elevated food reward sensitivity combines with diminished inhibitory control (Crane et al., 2023; Grol et al., 2022). These reward-driven eating behaviors are thought to involve overlapping neurobiological processes, particularly dopaminergic pathways and emotion regulation networks, which are themselves sensitive to stress-related modulation (Jastreboff et al., 2013; Berthoud, 2012; Sinha and Jastreboff, 2013; Adam and Epel, 2007).

Perceived stress has been associated with enhanced reward-seeking eating behavior and poorer self-regulation. However, the empirical evidence remains heterogeneous, particularly regarding associations with specific eating phenotypes and weight status (Sinha and Jastreboff, 2013; Adam and Epel, 2007; Kumar et al., 2022; Tryon et al., 2013). One potential reason for these inconsistencies is that stress is frequently conceptualized and measured as a unitary construct, despite representing a complex experience involving cognitive and affective appraisals of stressors including perceptions of control and coping capacity over demands and emotional distress (Chrousos and Gold, 1992). Accordingly, perceived stress is increasingly recognized as a multidimensional construct encompassing partially overlapping but functionally distinct psychological processes rather than representing a single homogenous construct. Collapsing stress into a single dimension may obscure potentially distinct stress-related processes that may differentially relate to reward-driven eating behavior.

The Perceived Stress Scale (PSS), widely used to assess subjective stress, differentiates two meaningful subscales: Helplessness, which reflects perceived uncontrollability over stressors, perceived inability to influence outcomes, and feeling overwhelmed by stressors; and Self-Efficacy, reflecting perceived coping capacity and confidence in one’s ability to manage stressful demands (Harris et al., 2023). Although both Helplessness and Self-Efficacy represent dimensions of perceived stress and are moderately related, they may have distinct behavioral consequences potentially differentially relevant in the context of reward-driven eating behavior.

Psychological models of stress-related eating propose that experiences of uncontrollability promote negative affect and emotional distress, which in turn increase motivation for rewarding behaviors, including consumption of highly palatable foods, that provide temporary affective relief (Sinha and Jastreboff, 2013; Adam and Epel, 2007). Consistent with this view, emotional and hedonic eating have frequently been conceptualized as emotion-regulation strategies whereby food-reward reduces unpleasant affect, consistent with negative reinforcement models of eating behavior (Tomiyama et al., 2011; Sinha and Jastreboff, 2013; Adam and Epel, 2007). Consequently, individuals who perceive stressors as uncontrollable may be more likely to engage in reward-driven eating as a means of regulating emotional distress (Sinha and Jastreboff, 2013; Adam and Epel, 2007; Hill et al., 2022).

Self-Efficacy reflects perceived confidence in one’s ability to cope with stressful demands (Harris et al., 2023; Klein et al., 2016; Cohen et al., 1983). Although higher stress related self-efficacy has generally been associated with more adaptive coping and better self-regulation, potentially associating with less reward-driven eating, lower self-efficacy with difficulties regulating behavior under stress, may increase vulnerability to reward-driven eating (Harris et al., 2023; Cohen et al., 1983). Thus, both Helplessness and Self-Efficacy may be associated with reward-driven eating. Although related, these dimensions reflect distinct aspects of stress and may therefore differ in the strength or pattern of their associations with reward-driven eating.

Despite the theoretical importance of the distinction between Helplessness and Self-Efficacy, most studies rely exclusively on the total PSS score, leaving it unclear whether these distinct stress dimensions differentially relate to reward-driven eating behavior. Disaggregating stress components may also help to clarify the inconsistent associations between perceived stress and body mass index (BMI), with positive, null, and inverse relationships reported (Singh et al., 2024; Huang et al., 2023; Shimanoe et al., 2015; Tilov et al., 2025). One possible explanation is that BMI is an indirect indicator of adiposity, and rather reflects long-term energy balance, which constrains the strength of cross-sectional associations (Tan et al., 2024; Sweatt et al., 2024). However, increasing evidence indicates that perceived stress may influence body weight indirectly via maladaptive eating patterns, particularly reward-driven eating (Sinha and Jastreboff, 2013; Wardle et al., 2011). Moreover, the specific stress-related mechanisms may differ according to BMI-defined weight status, given evidence for heightened reward sensitivity, altered stress reactivity, and diminished inhibitory control in obesity (Sinha and Jastreboff, 2013).

The present study addresses this gap by decomposing perceived stress into its control-related (Helplessness) and coping-related (Self-Efficacy) components and examining their independent associations with reward-driven eating behavior (hedonic hunger, trait food craving, and uncontrolled eating) in adults with BMI-defined normal weight and obesity.

We hypothesized that perceived stress would be associated with greater reward-driven eating. Furthermore, we hypothesized that both Helplessness and Self-Efficacy would be independently associated with reward-driven eating, but that the magnitude of these associations would differ, with stronger associations expected for Helplessness based on theoretical models emphasizing perceived uncontrollability in stress-related eating. Finally, we hypothesized that these associations would be stronger with increasing BMI and particularly among individuals with BMI-defined obesity as a clinically meaningful condition.

## 2 Materials and methods

### 2.1 Recruitment and study population

#### 2.1.1 Recruitment and eligibility

Participants were recruited as part of the Obese Taste Bud Study between 2020 and 2023 (NCT04633109 or DRKS00022950) through advertisements on the institutional homepage and flyers distributed within the campus of the University Clinic of Leipzig and the Helmholtz Institute for Metabolic, Obesity and Vascular Research (HI-MAG), Helmholtz Center Munich at the Leipzig University and Leipzig University Medical Center, Leipzig.

Potential participants were screened for eligibility in a telephone interview, during which a trained research assistant assessed inclusion and exclusion criteria: Male and female participants had to be aged between 18 and 69 years and were not included if they suffered from any severe kidney-, heart-, liver-, neurological- or mental disease, had been diagnosed with a malignant disease, had experienced radiation, chemotherapy or recent surgery.

#### 2.1.2 Analytical sample and missing data

For the present analyses, only participants with complete data on perceived stress (PSS) and covariates (BMI, age, sex, current hunger) were included (*N* = 90). Although ninety participants met the general analytical inclusion criteria. Although ninety participants met the general analytical inclusion criteria, the primary analysis sample comprised the 72 participants with normal weight or obesity.

Missing values on outcome variables (trait food craving, uncontrolled eating, and Power of Food scores) were retained to maximize sample size across analyses, and analyses were conducted using available cases for each outcome. In the primary analysis sample (*N* = 72 normal weight and obese), trait food craving was missing for one participant with normal weight and five participants with obesity. Uncontrolled eating was missing for four participants with obesity. PFS Total and PFS Availability were each missing for one participant with normal weight. Consequently, sample size varied between outcome-specific analyses; the corresponding N is reported for each regression model in Tables 1, 2, 3.

**Table 1:** Multiple linear regression analyses of associations between perceived stress and reward-driven eating outcomes in the primary analysis sample (*N* = 72).

**Outcome**	**Predictor**	***N***	***B* (95% CI)**	***β***	**Raw *p-*value**	**qFDR**	***R*** ^**2**^	**Adjusted *R*** ^**2**^	***F* (df)**	**Tolerance**	**VIF**
PFS Total	PSS Total	71	0.021 (−0.010; 0.052)	0.170	0.174	0.296	0.144	0.078	*F*(5,65) = 2.190	0.861	1.161
	PSS HL	**71**	**0.065 (0.020; 0.111)**	**0.356**	**0.006**	**0.017**	**0.222**	**0.149**	***F*(6,64) = 3.047**	**0.780**	**1.283**
	PSS SE	71	0.050 (−0.014; 0.114)	0.208	0.121	0.161	–	–	–	0.693	1.443
PFS availability	PSS Total	71	0.039 (0.005; 0.073)	0.262	0.027	0.135	0.247	0.189	*F*(5,65) = 4.259	0.861	1.161
	PSS HL	**71**	**0.077 (0.026; 0.129)**	**0.359**	**0.004**	**0.015**	**0.290**	**0.224**	***F*(6,64) = 4.360**	**0.780**	**1.283**
	PSS SE	71	0.024 (−0.048; 0.096)	0.085	0.506	0.506	–	–	–	0.693	1.443
PFS present	PSS Total	72	0.020 (−0.025; 0.065)	0.113	0.373	0.448	0.109	0.041	*F*(5,66) = 1.613	0.855	1.170
	PSS HL	72	0.068 (0.001; 0.135)	0.262	0.047	0.112	0.155	0.077	*F*(6,65) = 1.991	0.779	1.284
	PSS SE	72	0.058 (−0.035; 0.151)	0.170	0.220	0.240	–	–	–	0.687	1.455
PFS taste	PSS Total	72	0.003 (−0.034; 0.040)	0.022	0.861	0.861	0.079	0.010	*F*(5,66) = 1.137	0.855	1.170
	PSS HL	72	0.047 (−0.008; 0.103)	0.222	0.094	0.142	0.137	0.057	*F*(6,65) = 1.719	0.779	1.284
	PSS SE	72	0.068 (−0.009; 0.145)	0.244	0.084	0.142	–	–	–	0.687	1.455
UE	PSS Total	68	0.132 (0.003; 0.261)	0.257	0.045	0.135	0.164	0.097	*F*(5,62) = 2.437	0.857	1.167
	PSS HL	**68**	**0.335 (0.146; 0.523)**	**0.447**	**<0.001**	**0.009**	**0.261**	**0.188**	***F*(6,61) = 3.587**	**0.765**	**1.307**
	PSS SE	68	0.192 (−0.068; 0.452)	0.194	0.145	0.174	–	–	–	0.697	1.435
FC	PSS Total	66	0.396 (−0.211; 1.003)	0.162	0.197	0.296	0.209	0.143	*F*(5,60) = 3.168	0.854	1.171
	PSS HL	**66**	**1.434 (0.524; 2.345)**	**0.398**	**0.003**	**0.015**	**0.310**	**0.239**	***F*(6,59) = 4.409**	**0.735**	**1.361**
	PSS SE	66	1.179 (−0.039; 2.396)	0.254	0.057	0.115	–	–	–	0.681	1.469

Separate multiple linear regression models were estimated for PSS Total and for the PSS subscales Helplessness and Self-Efficacy. In the subscale models, Helplessness and Self-Efficacy were entered simultaneously. All models were adjusted for age, sex, BMI, and current hunger. Results are presented as unstandardized regression coefficients (B) with 95% confidence intervals, standardized regression coefficients (β), raw *p-*values, Benjamini–Hochberg FDR-adjusted *q* values, model *R*^2^ and adjusted *R*^2^, and overall model F statistics with numerator and denominator degrees of freedom. Tolerance and variance inflation factor (VIF) values are reported as collinearity diagnostics. FDR correction was applied across the six PSS Total coefficients and, separately, across the 12 coefficients comprising Helplessness and Self-Efficacy across the six outcomes. Results with *q* < 0.05 were considered statistically significant (bold). FC, trait food craving; FDR, false discovery rate; PFS, Power of Food Scale; PSS, Perceived Stress Scale; HL, Helplessness; SE, Self-Efficacy; UE, uncontrolled eating; VIF, variance inflation factor.

**Table 2:** Multiple linear regression analyses of associations between perceived stress and reward-driven eating outcomes in participants with obesity.

**Outcome**	**Predictor**	***N***	***B* (95% CI)**	***β***	**Raw p value**	**qFDR**	***R*** ^**2**^	**Adjusted *R*** ^**2**^	**Model *F* (df)**	**Tolerance**	**VIF**
PFS Total	PSS Total	25	**0.082 (0.023;0.140)**	**0.609**	**0.009**	**0.027**	**0.422**	**0.307**	***F*(4, 20) = 3.657**	**0.662**	**1.511**
	PSS HL	25	0.081 (0.013;0.149)	0.461	0.022	0.066	0.422	0.270	*F* (5, 19) = 2.780	0.887	1.127
	PSS SE	25	−0.084 (−0.218;0.051)	−0.328	0.208	0.312	-	-	-	0.480	2.085
PFS availability	PSS Total	25	**0.114 (0.048;0.179)**	**0.683**	**0.002**	**0.012**	**0.532**	**0.438**	***F*(4, 20) = 5.684**	**0.662**	**1.511**
	PSS HL	25	**0.113 (0.037;0.189)**	**0.518**	**0.006**	**0.036**	**0.532**	**0.409**	***F*(5, 19) = 4.320**	**0.887**	**1.127**
	PSS SE	25	−0.116 (−0.266;0.035)	−0.365	0.124	0.219	-	-	-	0.480	2.085
PFS present	PSS Total	25	0.033 (−0.049;0.115)	0.199	0.413	0.413	0.248	0.097	*F*(4, 20) = 1.648	0.662	1.511
	PSS HL	25	0.042 (−0.053;0.138)	0.195	0.366	0.488	0.255	0.059	*F* (5, 19) = 1.302	0.887	1.127
	PSS SE	25	0.002 (−0.187;0.191)	0.006	0.984	0.984	-	-	-	0.480	2.085
PFS taste	PSS Total	25	**0.082 (0.017;0.148)**	**0.568**	**0.016**	**0.027**	**0.377**	**0.253**	***F*(4, 20) = 3.027**	**0.662**	**1.511**
	PSS HL	25	0.074 (−0.002;0.150)	0.388	0.056	0.134	0.385	0.223	*F* (5, 19) = 2.378	0.887	1.127
	PSS SE	25	−0.114 (−0.264; 0.036)	−0.413	0.128	0.219	-	-	-	0.480	2.085
UE	PSS Total	21	**0.385 (0.076;0.693)**	**0.649**	**0.018**	**0.027**	**0.374**	**0.217**	***F*(4, 16) = 2.389**	**0.649**	**1.540**
	PSS HL	21	**0.481 (0.138;0.823)**	**0.691**	**0.009**	**0.036**	**0.437**	**0.249**	***F*(5, 15) = 2.328**	**0.877**	**1.140**
	PSS SE	21	−0.024 (−0.691;0.643)	−0.021	0.940	0.984	-	-	-	0.479	2.088
FC	PSS Total	20	**1.292 (0.101;2.484)**	**0.522**	**0.035**	**0.042**	**0.508**	**0.376**	***F*(4, 15) = 3.866**	**0.644**	**1.552**
	PSS HL	20	**1.792 (0.549;3.035)**	**0.552**	**0.008**	**0.036**	**0.608**	**0.468**	**F(5, 14) = 4.348**	**0.877**	**1.141**
	PSS SE	20	0.638 (−1.809;3.079)	0.136	0.586	0.703	-	-	-	0.470	2.129

Separate multiple linear regression models were estimated for PSS Total and for Helplessness and Self-Efficacy entered simultaneously. Models were adjusted for age, sex, and current hunger. BMI was not included as a covariate because BMI defined the stratification by obesity status. Results are presented as unstandardized regression coefficients (B) with 95% confidence intervals, standardized regression coefficients (β), raw *p-*values, Benjamini–Hochberg FDR-adjusted *q* values, model *R*^2^ and adjusted *R*^2^, and overall model F statistics with numerator and denominator degrees of freedom. Tolerance and variance inflation factor (VIF) values are reported as collinearity diagnostics. FDR correction was applied across the six PSS Total coefficients and, separately, across the 12 Helplessness and Self-Efficacy coefficients. Results with *q* < 0.05 were considered statistically significant (bold). FC, trait food craving; FDR, false discovery rate; PFS, Power of Food Scale; PSS, Perceived Stress Scale; HL, Helplessness; SE, Self-Efficacy; UE, uncontrolled eating; VIF, variance inflation factor.

**Table 3:** Multiple linear regression analyses of associations between perceived stress and reward-driven eating outcomes in participants with normal weight.

**Outcome**	**Predictor**	***N***	***B* (95% CI)**	***β***	**Raw *p-*value**	**qFD*R***	***R*** ^**2**^	**Adjusted *R*** ^**2**^	**Model *F* (df)**	**Tolerance**	**VIF**
PFS total	PSS Total	46	−0.001 (−0.038; 0.037)	−0.005	0.973	0.973	0.107	0.020	*F*(4,41) = 1.229	0.936	1.068
	PSS HL	46	0.065 (−0.002; 0.132)	0.347	0.059	0.193	0.212	0.114	*F*(5,40) = 2.154	0.620	1.614
	PSS SE	46	0.088 (0.004; 0.173)	0.362	0.041	0.193	–	–	–	0.669	1.494
PFS availability	PSS Total	46	0.013 (−0.028; 0.055)	0.099	0.521	0.973	0.103	0.016	*F*(4,41) = 1.178	0.936	1.068
	PSS HL	46	0.071 (−0.007; 0.148)	0.339	0.072	0.193	0.168	0.064	*F*(5,40) = 1.612	0.620	1.614
	PSS SE	46	0.064 (−0.034; 0.161)	0.233	0.193	0.290	–	–	–	0.669	1.494
PFS present	PSS Total	47	0.012 (−0.045; 0.068)	0.066	0.678	0.973	0.036	−0.056	*F*(4,42) = 0.393	0.928	1.078
	PSS HL	47	0.088 (−0.017; 0.193)	0.320	0.097	0.193	0.102	−0.008	*F*(5,41) = 0.931	0.619	1.616
	PSS SE	47	0.090 (−0.041; 0.221)	0.253	0.171	0.290	–	–	–	0.666	1.501
PFS taste	PSS Total	47	−0.025 (−0.065; 0.015)	−0.171	0.212	0.973	0.292	0.224	*F*(4,42) = 4.322	0.928	1.078
	PSS HL	47	0.034 (−0.039; 0.107)	0.149	0.356	0.356	0.349	0.270	*F*(5,41) = 4.397	0.619	1.616
	PSS SE	47	0.103 (0.012; 0.194)	0.351	0.028	0.193	–	–	–	0.666	1.501
UE	PSS Total	47	0.062 (−0.081; 0.205)	0.136	0.388	0.973	0.052	−0.039	*F*(4,42) = 0.572	0.928	1.078
	PSS HL	47	0.234 (−0.034; 0.502)	0.331	0.086	0.193	0.102	−0.007	*F*(5,41) = 0.936	0.619	1.616
	PSS SE	47	0.167 (−0.167; 0.501)	0.183	0.319	0.348	–	–	–	0.666	1.501
FC	PSS Total	46	0.066 (−0.671; 0.804)	0.028	0.857	0.973	0.093	0.005	*F*(4,41) = 1.055	0.920	1.087
	PSS HL	46	0.863 (−0.648; 2.374)	0.231	0.255	0.340	0.126	0.017	*F*(5,40) = 1.151	0.546	1.832
	PSS SE	46	0.910 (−0.867; 2.687)	0.195	0.307	0.348	–	–	–	0.616	1.623

Separate multiple linear regression models were estimated for PSS Total and for Helplessness and Self-Efficacy entered simultaneously. The normal-weight subgroup comprised participants with BMI 18.5–24.9 kg/m^2^. Models were adjusted for age, sex, and current hunger; BMI was not included because the subgroup was defined according to BMI. Results are presented as unstandardized regression coefficients (B) with 95% confidence intervals, standardized regression coefficients (β), raw *p* values, Benjamini–Hochberg FDR-adjusted *q* values, model R^2^ and adjusted *R*^2^, and overall model *F* statistics with numerator and denominator degrees of freedom. Tolerance and variance inflation factor (VIF) values are reported as collinearity diagnostics. FDR correction was applied across the six PSS Total coefficients and, separately, across the 12 Helplessness and Self-Efficacy coefficients. Results with *q* < 0.05 were considered statistically significant (bold). FC, trait food craving; FDR, false discovery rate; PFS, Power of Food Scale; PSS, Perceived Stress Scale; HL, Helplessness; SE, Self-Efficacy; UE, uncontrolled eating; VIF, variance inflation factor.

### 2.2 Study design

The study design is described in greater detail elsewhere (Kersten et al., 2024). Briefly, participants arrived at the outpatient unit of the University Clinic of Leipzig or the HI-MAG at 7:30 o’clock in the morning after an overnight fast (>12 h). Upon arrival, informed written consent was obtained. All procedures met the standards of the Declaration of Helsinki and were approved by the ethics committee of the Medical Faculty of the University of Leipzig, Leipzig (protocol code 011/20-ek).

Participants´ characteristics, medical and behavioral data were assessed during a standardized interview and through self-report questionnaires. Amongst others, the following information was collected: Current dieting was assessed via self-report as a dichotomous variable (1 = yes, 0 = no). Current hunger was assessed on a visual analogue scale anchored with 0 (not hungry) and 100 (very hungry). Current smoking was assessed as a dichotomous variable (1 = yes, 0 = no), defined as smoking at least one cigarette per day. Furthermore, the following validated questionnaires were applied.

#### 2.2.1 Perceived stress scale

Perceived stress was assessed using the 10-item Perceived Stress Scale (PSS), which evaluates the extent to which individuals appraise situations in their lives as stressful (Cohen et al., 1983). Items are rated on a 5-point Likert scale from 1 (“never”) to 5 (“very often”).

Following the established two-factor scoring procedure, the Helplessness subscale was calculated as the sum of items 1, 2, 3, 6, 9, and 10 (possible range 6–30). The Self-Efficacy subscale was calculated as the sum of items 4, 5, 7, and 8 in their original direction (possible range 4–20); these items were not reverse-scored for the subscale, so higher scores indicate greater perceived self-efficacy. For the PSS Total score, items 4, 5, 7, and 8 were reverse-scored, and all 10 items were summed (possible range 10–50), with higher values indicating greater perceived stress (Klein et al., 2016; Roberti et al., 2006).

The PSS has demonstrated good internal consistency (Cronbach’s alpha for the PSS Total score, 0.78–0.91; subscale alpha values ≥0.81) and validity across diverse populations (Harris et al., 2023; Klein et al., 2016; Cohen et al., 1983; Roberti et al., 2006).

#### 2.2.2 Power of food scale

The Power of Food Scale (PFS) assesses individual differences in hedonic hunger (Lowe et al., 2009). Fifteen items must be answered on a 5-point Likert scale from 1 (I do not agree at all) to 5 (I strongly agree). Three subscales further differentiate the proximity of food to people necessary to trigger appetitive motivation. Food availability assesses the appetitive motivation when food is constantly available. Food present and food taste assess appetitive motivation when food is present or has already been tasted, respectively. Higher scores signal greater appetitive motivation and less subjective control in response to food-abundant environments. Cronbach’s alpha was 0.92 for the total scale, 0.85 for the Present- and Availability subscale and 0.80 for the taste subscale (Andreeva et al., 2019).

#### 2.2.3 Food craving questionnaire- trait reduced version

The Food Craving Questionnaire – trait reduced version (FCQ-T-r) assesses the general tendency to experience food craving (Meule et al., 2014). This 15-item self-report instrument measures the frequency and intensity of food craving as a stable trait. Items are rated on a 5-point Likert scale from 1 (never) to 5 (always), with higher scores indicating stronger trait-like food cravings. It has demonstrated excellent internal consistency (Cronbach’s alpha 0.90) and good construct validity in non-clinical populations (Meule et al., 2014).

#### 2.2.4 Three-factor eating questionnaire

The German version of the 51-item Three-Factor Eating Questionnaire (TFEQ) was applied to assess uncontrolled eating behavior (Stunkard and Messick, 1985; Pudel and Westenhöfer, 1989). All items are coded with 0 or 1. Scoring followed the same procedure as proposed in the original instrument where higher scores indicate greater uncontrolled eating tendencies (Stunkard and Messick, 1985). The subscale “uncontrolled eating” was formed based on the factor structure identified in a large validation study of the German version of the TFEQ (Löffler et al., 2015). Cronbach’s alpha for uncontrolled eating was 0.80 (Löffler et al., 2015).

#### 2.2.5 International physical activity questionnaire

Physical activity was assessed using the International Physical Activity Questionnaire (IPAQ), which captures self-reported walking, moderate- and vigorous physical activity over the previous 7 days. Based on established scoring guidelines responses were classified into low, moderate, or high physical activity categories. The IPAQ has demonstrated acceptable reliability (test–retest correlations: 0.76–0.97) and validity (correlations: 0.30–0.40) across multiple countries (Craig et al., 2003).

### 2.3 Anthropometric measures

Body weight and height were measured using a calibrated scale with a stadiometer (Seca, Hamburg, Germany) without shoes to the nearest 0.5 kg and 0.1 cm, respectively. Body mass index (BMI) was calculated as body weight (kg) divided by body height squared (m^2^). Participants were classified according to BMI as having normal weight (BMI 18.5–24.9 kg/m^2^), overweight (BMI 25.0–29.9 kg/m^2^), or obesity (BMI ≥ 30 kg/m^2^) according to World Health Organization criteria (World Health Organization, 2000).

### 2.4 Statistical analysis

Data were analyzed using SPSS version 29 (IBM, Ehningen, Germany) and GraphPad Prism version 10 (GraphPad Software, San Diego, USA). Descriptive data are reported as median and interquartile range (IQR). For the primary group comparison, participants with BMI-defined normal weight (BMI 18.5–24.9 kg/m^2^) were compared with participants with obesity (BMI ≥ 30 kg/m^2^). Participants with overweight (BMI 25.0–29.9 kg/m^2^) were excluded from the primary inferential group comparisons but retained descriptively in Table 4. Continuous variables were compared between the normal-weight and obesity groups using Mann–Whitney *U* tests. Sex was compared using Pearson’s chi-square test. Because of sparse expected cell frequencies, current smoking and current dieting were compared using Fisher’s exact tests. Physical activity was compared using the Fisher–Freeman–Halton exact test with Monte Carlo estimation of the two-sided *p-*value. Effect sizes were reported as r for Mann–Whitney *U* tests and Cramér’s *V* for categorical comparisons.

**Table 4:** Characteristics of the study population stratified by BMI-defined weight status.

Characteristic	**Sample *N* = 90**	**Primary analysis sample *N* = 72**	***N* = 47 (52.2%) with** **normal weight**	***N* = 18 (20%) with overweight**	***N* = 25 (27.8%) with obesity**	**Statistical test**	***p*-value**	**Effect size**	**FDR *q***
Body weight (kg)	75.45 (24.35)	74.90 (29.70)	63.00 (17.40)	77.85 (11.23)	105.00 (33.00)	*U* = 1156.0	<0.001	*r* = 0.79	–
Height (cm)	171.50 (12.85)	171.50 (13.53)	173.00 (15.00)	171.00 (11.00)	169.00 (11.95)	*U* = 486.0	0.230	*r* = 0.14	–
BMI (kg/m^2^)	24.60 (8.70)	23.52 (11.81)	22.23 (2.98)	26.72 (2.03)	35.71 (8.39)	*U* = 1175.0	<0.001	*r* = 0.82	–
Sex (female/male), *N* (%)	62 (68.9)/ 28 (31.1)	49 (68.1) / 23 (31.9)	32 (68.1)/ 15 (31.90)	13 (72.2)/ 5 (27.8)	17 (68.00)/ 8 (32.00)	χ^2^(1) < 0.001	0.994	Cramér’s *V* = 0.001	–
Age (years)	33.00 (23.25)	31.00 (24.50)	28.00 (20.00)	37.00 (22.50)	42.00 (25.00)	*U* = 832.0	0.004	*r* = 0.34	–
Smoking N = yes/no (%)	9 (10.00)/81(90.00)	8 (11.1) / 64 (88.9)	4 (8.50)/ 43 (91.50)	1 (5.6)/ 17 (94.4)	4 (16%)/ 21 (84%)	Fisher exact	0.436	Cramér’s *V* = 0.11	–
Current hunger (mm)	30.00 (40.00)	35.00 (30.00)	40.00 (20.00)	25.00 (45.00)	20.00 (45.00)	*U* = 460.5	0.130	*r* = 0.18	–
Current dieting N = yes/no (%)	10 (11.10)/ 80 (88.90)	7 (9.7) / 65 (90.3)	2 (4.30)/ 45 (95.70)	3 (16.70)/ 15 (83.30)	5 (20.00)/ 20 (80.00)	Fisher exact	0.045	Cramér’s *V* = 0.25	–
Physical activity (%)						FFH exact (Monte Carlo)	0.036	Cramér’s *V* = 0.33	–
Low	5 (6.0%)	4 (6.1)	0 (0%)	1 (5.9%)	4 (16.0%)		–	–	–
Moderate	5 (6.0%)	4 (6.1)	3 (7.3%)	1 (5.9%)	1 (4.0%)		–	–	–
High	73 (88.0%)	58 (87.9%)	38 (92.7%)	15 (88.2%)	20 (80.0%)		–	–	–
PSS Total score	24.00 (9.00)	24.00 (8.50)	23.00 (9.00)	22.00 (14.50)	25.00 (6.50)	*U* = 651.5	0.448	*r* = 0.09	0.576
PSS Helplessness	14.00 (6.00)	14.50 (5.75)	14.00 (5.00)	13.00 (10.25)	15.00 (6.00)	*U* = 532.5	0.513	*r* = 0.08	0.577
PSS Self-Efficacy	15.00 (4.00)	15.00 (4.00)	16.00 (4.00)	15.00 (4.25)	14.00 (3.50)	*U* = 388.5	0.018	*r* = 0.28	0.095
Trait food craving	31.00 (16)	30.00 (16.25)	28.50 (14.00)	35.00 (19.00)	34.00 (16.50)	*U* = 601.0	0.049	*r* = 0.24	0.144
PFS Total score	2.40 (0.94)	2.40 (0.93)	2.30 (0.80)	2.40 (1.05)	2.53 (1.03)	*U* = 673.5	0.235	*r* = 0.14	0.353
PFS Availability	1.83 (1.00)	1.83 (1.00)	1.67 (0.67)	1.67 (1.50)	2.00 (1.58)	*U* = 706.5	0.112	*r* = 0.19	0.202
PFS Present	2.25 (1.50)	2.00 (1.25)	1.75 (1.25)	3.00 (1.31)	2.50 (1.25)	*U* = 781.0	0.021	*r* = 0.27	0.095
PFS Taste	3.00 (1.40)	3.00 (1.40)	3.00 (1.40)	2.80 (1.60)	2.80 (1.30)	*U* = 547.5	0.635	*r* = 0.06	0.635
Trait uncontrolled eating	4.00 (5.00)	4.00 (4.75)	4.00 (2.00)	5.00 (5.50)	5.00 (5.50)	*U* = 631.5	0.064	*r* = 0.22	0.144

Data are presented as median (interquartile range) for continuous variables and n (%) for categorical variables. The overweight subgroup is retained descriptively but excluded from the primary inferential group comparison. Continuous variables in the primary normal-weight versus obesity comparison were compared using Mann–Whitney *U* tests; effect sizes are reported as *r* = |z|/√N. Sex was compared using Pearson’s chi-square test. Smoking and current dieting were compared using Fisher’s exact tests because of sparse expected cell frequencies. Physical activity was compared using the Fisher–Freeman–Halton exact test with Monte Carlo estimation of the two-sided *p* value because of sparse expected cell frequencies. Cramér’s *V* is reported for categorical comparisons. For the nine perceived-stress and reward-driven eating variables, *p-*values were adjusted using the Benjamini–Hochberg false discovery rate procedure; *q* < 0.05 was considered statistically significant. FDR correction was not applied to general participant characteristics. Physical activity was missing for six participants with normal weight and one participant with overweight. Trait food craving was missing for one participant with normal weight, three participants with overweight, and five participants with obesity. Trait uncontrolled eating was missing for one participant with overweight and four participants with obesity. PFS Total and PFS Availability were missing for one participant with normal weight. BMI, body mass index; FDR, false discovery rate; FFH, Fisher–Freeman–Halton; PFS, Power of Food Scale; PSS, Perceived Stress Scale.

For the nine perceived-stress and reward-driven eating variables (PSS Total, Helplessness, Self-Efficacy, PFS Total, PFS Availability, PFS Present, PFS Taste, trait food craving, and uncontrolled eating), *p* values from the Mann–Whitney *U* tests comparing participants with normal weight and obesity were additionally adjusted for multiple testing using the Benjamini–Hochberg false discovery rate procedure; *q* < 0.05 was considered statistically significant. FDR correction was not applied to general participant characteristics.

Associations between perceived stress, eating behavior, and participants´ characteristics were examined using Spearman’s rank correlation.

Associations between perceived stress and reward-driven eating outcomes were examined using multiple linear regression analyses. Separate models were estimated for the PSS Total score and for the PSS subscales. For models including the PSS subscales, Helplessness and Self-Efficacy were entered simultaneously as predictors to estimate their independent associations with each outcome while accounting for their shared variance. All regression models were adjusted for age, sex, and current hunger, with BMI additionally included as a covariate in analyses of the primary analysis sample. In the BMI-defined subgroup analyses conducted separately among participants with normal weight and participants with obesity, BMI was not included as a covariate because obesity status was used to define the analytical groups. Hedonic hunger (Power of Food Scale Total score and subscales), trait food craving (Food Craving Questionnaire-trait reduced), and uncontrolled eating (Three-Factor Eating Questionnaire Uncontrolled Eating score) were analyzed as dependent variables.

Sensitivity analyses were performed to examine whether the primary associations were robust to additional adjustment for current smoking, physical activity and current dieting. The primary linear regression models were repeated with current smoking and current dieting added as a binary covariate and, separately, with physical activity added using indicator variables for low and moderate activity, with high activity as the reference category. In the primary analysis sample, models were adjusted for age, sex, BMI, and current hunger in addition to the respective sensitivity covariates. In analyses stratified by obesity status, BMI was not included because it defined the analytical groups; these models were adjusted for age, sex, and current hunger in addition to the respective sensitivity covariates. PSS Total was examined in separate models, whereas Helplessness and Self-Efficacy were entered simultaneously in the subscale models (Supplementary Tables S6–S8).

To assess multicollinearity particularly between the PSS Helplessness and Self-Efficacy subscales, variance inflation factor (VIF) values and tolerance values were examined for all primary and subgroup regression models. Exact model-specific values are reported in Tables 1–3. No model showed evidence of problematic multicollinearity (all VIFs < 5.0; all tolerance values > 0.20).

Multiple testing was controlled using the Benjamini–Hochberg false discovery rate (FDR) procedure (*q* < 0.05). FDR correction was applied separately within each sample (primary analysis sample, normal-weight and obesity subgroup) across the six outcomes for PSS Total models and across the 12 primary regression coefficients (Helplessness and Self-Efficacy × six outcomes) for PSS subscale models.

To formally test whether associations between perceived stress and reward-driven eating differed according to BMI and BMI-defined weight status, separate moderation analyses were conducted in the primary analysis sample using the PROCESS macro version 4.2 beta (Model 1). Participants with overweight were excluded from these analyses. Separate models were estimated with PSS Total, PSS Helplessness, or PSS Self-Efficacy as the focal predictors (Hayes, 2022), and each reward-driven eating behavior outcome (PFS Total score, PFS subscales, trait food craving, and uncontrolled eating) as the dependent variable. Age, sex, and current hunger were included as covariates. In models with PSS Helplessness or PSS Self-Efficacy as the focal predictor, the other PSS subscale was additionally included to account for their shared variance.

Two complementary moderation approaches were applied in the primary analysis sample. First, BMI was entered as a continuous moderator to examine whether associations varied across the observed BMI range. Second, BMI-defined weight status (normal-weight: BMI 18.5–24.9 kg/m^2^; obesity: BMI ≥ 30 kg/m^2^) was entered as a categorical moderator to evaluate whether the observed associations differed between these two groups. Participants with overweight were excluded from both moderation analyses. BMI and the respective continuous focal PSS predictor were mean-centered before calculation of the interaction terms; in models using categorical weight status, the focal PSS predictor was mean-centered. Interaction effects were evaluated using heteroskedasticity-consistent (HC3) standard errors. To account for multiple testing across moderation models, FDR correction was applied to all interaction-term *p*-values.

As this study was a retrospective analysis of data collected for a different primary objective, no *a priori* power calculation was performed. Instead, sample size was determined by data availability.

To aid interpretation of the findings, *post hoc* sensitivity power analyses using G*Power 3.1 were conducted (Erdfelder et al., 2009). Assuming α = 0.05 and a primary analysis sample of *N* = 72, the multiple regression models including perceived stress and the covariates age, sex, BMI, and current hunger had 80% power to detect effects of approximately *f*^2^ = 0.11, corresponding to small-to-moderate effect sizes. Statistical power was more limited in the BMI-defined subgroup analyses: in the normal-weight subgroup (*N* = 47) effects of approximately *f*^2^ = 0.18 were detectable with 80% power, whereas in the obesity subgroup (*N* = 25), only effects with approximately *f*^2^ = 0.35 were detectable. For the moderation analyses conducted using PROCESS Model 1, including perceived stress, BMI-defined weight status or BMI, their interaction term, and the covariates age, sex, and current hunger, the study had 80% power to detect interaction effects of approximately *f*^2^ = 0.11–0.12.

## 3 Results

### 3.1 Description of the study population

Participants were mostly female (68.9%) and aged 20 to 69 years. Of the 90 participants, 47 (52.2%) had normal weight, 18 (20.0%) had overweight, and 25 (27.8%) had obesity (Table 4). Participants with overweight were excluded from the primary inferential analyses, resulting in a primary analysis sample of *N* = 72 comprising 47 participants with normal weight and 25 participants with obesity. Body weight and, by definition, BMI differed significantly between participants with normal weight and obesity. Age also differed between groups with normal weight and obesity (*p* = 0.004). Participants with obesity were older than participants with normal weight. Current dieting was more frequent among participants with obesity than among participants with normal weight (*p* = 0.045). Physical activity also differed between groups (*p* = 0.036), with low physical activity occurring only among participants with obesity.

None of the perceived stress or reward-driven eating variables differed significantly between the normal-weight and obesity groups after FDR correction. Nominal group differences were observed for PSS Self-Efficacy, PFS Present, and trait food craving, but did not remain significant after FDR correction (all *q* ≥ 0.095). No other perceived stress or reward-driven eating variable differed significantly between participants with normal weight and obesity.

### 3.2 Association of perceived stress, measures of food reward, and BMI

PSS Total and subscale scores were not significantly associated with BMI in the primary analysis sample or in the normal-weight or obese subsample (*p* > 0.05; Supplementary Tables S1–S3). In the primary analysis sample, only the PFS Availability and Present subscales correlated positively with BMI, while no PFS indices were significantly associated with BMI in the subsample having obesity or in the normal weight subsample. Trait food craving and uncontrolled eating were positively associated with BMI in the primary analysis sample, but not in the normal weight group or the group with obesity.

As presented in Table 5, in the primary analysis sample, PSS Total scores correlated positively with PFS Total, PFS Availability and PFS Present subscale.

**Table 5:** Association of perceived stress scores and measures of reward-driven eating behavior in the primary analysis sample and in participants with normal weight or obesity.

Sample		Power of food scale						Perceived stress scale	
	Perceived stress scale	Total score	Availability	Present	Taste	Food craving	Uncontrolled eating	Total	Helplessness
Primary analysis sample *N* = 72	Total score	***r* = 0.241** ***p* = 0.043**	***r* = 0.404** ***p* < 0.001**	***r* = 0.292** ***p* = 0.013**	*r* = 0.052 *p* = 0.662	***r* = 0.253** ***p* = 0.040**	*r* = 0.186 *p* = 0.129	–	–
	Helplessness	***r* = 0.315** ***p* = 0.007**	***r* = 0.415** ***p* < 0.001**	***r* = 0.300** ***p* = 0.010**	*r* = 0.196 *p* = 0.100	***r* = 0.324** ***p* = 0.008**	***r* = 0.280** ***p* = 0.021**	***r* = 0.847** ***p* < 0.001**	–
	Self-efficacy	*r* = −0.028 *p* = 0.817	*r* = −0.207 *p* = 0.083	*r* = −0.157 *p* = 0.187	*r* = 0.122 *p* = 0.309	*r* = −0.140 *p* = 0.261	*r* = −0.045 *p* = 0.717	***r* = −0.825** ***p* < 0.001**	***r* = −0.444** ***p* < 0.001**
*N* = 47 no*r*mal weight	Total score	*r* = 0.121 *p* = 0.423	***r* = 0.295** ***p* = 0.046**	*r* = 0.249 *p* = 0.092	*r* = −0.076 *p* = 0.613	*r* = 0.240 *p* = 0.109	*r* = 0.169 *p* = 0.255	–	–
	Helplessness	***r* = 0.294** ***p* = 0.047**	***r* = 0.401** ***p* = 0.006**	***r* = 0.330** ***p* = 0.023**	*r* = 0.064 *p* = 0.671	***r* = 0.314** ***p* = 0.034**	*r* = 0.227 *p* = 0.125	***r* = 0.904** ***p* < 0.001**	–
	Self-efficacy	*r* = 0.125 *p* = 0.408	*r* = −0.087 *p* = 0.566	*r* = −0.084 *p* = 0.573	*r* = 0.236 *p* = 0.110	*r* = −0.110 *p* = 0.468	*r* = −0.022 *p* = 0.883	***r* = −0.847** ***p* < 0.001**	***r* = −0.560** ***p* < 0.001**
*N* = 25 with obesity	Total sco*r*e	***r* = 0.502** ***p* = 0.011**	***r* = 0.655** ***p* < 0.001**	***r* = 0.424** ***p* = 0.035**	*r* = 0.304 *p* = 0.140	*r* = 0.357 *p* = 0.122	*r* = 0.298 *p* = 0.189	–	–
	Helplessness	***r* = 0.417** ***p* = 0.038**	***r* = 0.567** ***p* = 0.003**	*r* = 0.360 *p* = 0.077	*r* = 0.319 *p* = 0.120	***r* = 0.474** ***p* = 0.035**	***r* = 0.515** ***p* = 0.017**	***r* = 0.783** ***p* < 0.001**	–
	Self-efficacy	*r* = −0.300 *p* = 0.145	***r* = −0.411** ***p* = 0.041**	*r* = −0.232 *p* = 0.264	*r* = −0.199 *p* = 0.340	*r* = −0.182 *p* = 0.443	*r* = −0.029 *p* = 0.899	***r* = −0.774** ***p* < 0.001**	*r* = −0.328 *p* = 0.110

Spearman’s rank correlation coefficient was used to assess associations between perceived stress and Power of Food scores, trait food craving and trait uncontrolled eating in the primary analysis sample (normal weight + obesity) and separately in participants with normal weight or obesity. Results are presented as correlation coefficient (*r*) with *p*-values. A *p*-value < 0.05 was considered statistically significant (bold). Trait uncontrolled eating was assessed with the Three -Factor Eating Questionnaire. Trait food craving was assessed with the Food Craving Questionnaire-trait reduced. Data were missing for trait food craving in five participants with obesity and one participant with normal weight, for uncontrolled eating in four participants with obesity, and for PFS Total and PFS Availability in one participant with normal weight. N, number.

PSS Helplessness was associated with PFS Total, PFS Availability, and PFS Present, whereas PSS Self-Efficacy was not significantly related to any PFS measure. Among individuals with obesity, PSS Total scores correlated with PFS Total, Availability, and Present scores and PSS Helplessness with PFS Total and Availability scores. Self-Efficacy was negatively correlated with PFS Availability in this subgroup. Among normal-weight participants, PSS Total scores were related only to PFS Availability, and PSS Helplessness to PFS Total, PFS Availability, and PFS Present scores.

In the primary analysis sample, PSS Total scores were positively correlated with trait food craving. PSS Helplessness was positively associated with both food craving and uncontrolled eating, whereas PSS Self-Efficacy was unrelated to either outcome. In the subgroup with obesity, PSS Helplessness correlated with both trait food craving and uncontrolled eating. In normal-weight participants, PSS Helplessness was associated only with trait food craving, whereas PSS Total was unrelated to either outcome.

Across all samples, PSS Total was strongly positively correlated with PSS Helplessness (all *p* < 0.001) and strongly negatively correlated with PSS Self-Efficacy (all *p* < 0.001). PSS Helplessness and PSS Self-Efficacy were inversely related in all samples, although this association reached statistical significance only in the primary analysis sample and among normal-weight participants. Despite the moderate inverse correlation between Helplessness and Self-Efficacy, multicollinearity diagnostics indicated no evidence of problematic multicollinearity (all VIFs < 5.0 and tolerance values > 0.20).

### 3.3 Perceived stress associates with measures of food reward in multiple linear regression analyses

#### 3.3.1 Primary analysis sample

Multiple linear regression results are presented in Table 1. In the primary analysis sample, higher PSS Total scores were not independently associated with any reward-driven eating outcome after adjusting for age, sex, BMI, and current hunger and following FDR correction. Nominal positive associations were observed with PFS Availability (*p* = 0.027) and uncontrolled eating (*p* = 0.045), but neither remained significant after FDR correction (both *q* = 0.135).

In models in which both PSS Helplessness and PSS Self-Efficacy were entered simultaneously, higher PSS Helplessness scores were independently associated with higher PFS Total and Availability scores, as well as greater trait food craving and uncontrolled eating, after adjustment for age, sex, BMI, and current hunger (all FDR corrected). Associations with PFS Present and PFS Taste did not remain significant after FDR correction. In contrast, PSS Self-Efficacy was not independently associated with any reward-driven eating measure after FDR correction.

#### 3.3.2 Subgroup with obesity

In individuals with obesity (Table 2), higher PSS Total scores were independently associated with higher PFS Total, Availability, and Taste scores, as well as greater trait food craving and uncontrolled eating, after covariate adjustment and FDR correction.

In models in which both PSS Helplessness and PSS Self-Efficacy were entered simultaneously, higher PSS Helplessness scores were independently associated with higher PFS Availability scores, and greater trait food craving and uncontrolled eating, after adjustment for age, sex, and current hunger (FDR corrected). Associations of PSS Helplessness with PFS Total and Taste scores did not remain significant after FDR correction. PSS Self-Efficacy was not independently associated with any reward-driven eating measure after FDR correction.

#### 3.3.3 Subgroup with normal weight

Among participants with normal weight, PSS Total scores were not independently associated with any measure of reward-driven eating after adjustment for age, sex, and current hunger and FDR correction (Table 3). In models in which both PSS Helplessness and PSS Self-Efficacy were entered simultaneously, neither PSS Helplessness nor PSS Self-Efficacy was independently associated with any reward-driven eating measure after FDR correction. Nominal positive associations were observed between PSS Self-Efficacy and PFS Total (*p* = 0.041) and PFS Taste (*p* = 0.028), but neither remained statistically significant after FDR correction.

#### 3.3.4 Sensitivity analyses

Sensitivity analyses with additional adjustment for current smoking, physical activity, or current dieting supported the robustness of the main findings (Supplementary Tables S6–S8). Overall, additional adjustment for these variables did not materially alter the pattern of associations observed in the primary regression models. Effect directions and magnitudes were generally comparable in the primary analysis sample and the BMI-defined subgroups, although individual coefficient estimates and raw *p* values showed some variation. In particular, some associations were attenuated in the smaller subgroup analyses. In the primary analysis sample, the independent associations of PSS Helplessness with PFS Total, PFS Availability, trait food craving, and uncontrolled eating remained significant after separate additional adjustment for current smoking, physical activity, and current dieting. Overall, the sensitivity analyses supported the robustness of the central Helplessness findings to additional adjustment for these potential confounding factors.

### 3.4 Moderation of stress-reward-driven eating associations by BMI and weight status

Moderation analyses examined whether BMI or weight status (normal weight vs. obesity) influenced associations between perceived stress and reward-driven eating outcomes within the primary analysis sample, after adjustment for age, sex and current hunger. In models with PSS Helplessness or PSS Self-Efficacy as the focal predictor, the other PSS subscale was included as an additional covariate (Supplementary Tables S4, S5).

When weight status was examined as a moderator, no interaction effects remained statistically significant after correction for multiple testing using the FDR procedure. Before FDR correction, nominal interaction effects were observed for the interactions between weight status and PSS Total score for PFS Availability (*b* = 0.092, SE = 0.033, *p* = 0.006, *q* = 0.112), and between weight status and PSS Self-Efficacy predicting PFS Availability (*b* = −0.188, SE = 0.085, *p* = 0.031, *q* = 0.173), No other weight status interaction effects showed nominal significance.

Using BMI as a continuous moderator, no interaction effects remained statistically significant after FDR correction. Before FDR correction, nominal interaction effects were observed between BMI and PSS Total for PFS Total (*b* = 0.0047, SE = 0.0021, *p* = 0.031), PFS Availability (*b* = 0.0057, SE = 0.0018, *p* = 0.003), and PFS Taste (*b* = 0.0044, SE = 0.0020, *p* = 0.028). Nominal interactions were also observed between BMI and PSS Self-Efficacy for PFS Total (*b* = −0.0088, SE = 0.0043, *p* = 0.046), PFS Availability (*b* = −0.0096, SE = 0.0044, *p* = 0.031), and PFS Taste (*b* = −0.0092, SE = 0.0043, *p* = 0.034). No interaction involving PSS Helplessness reached nominal statistical significance in the continuous-BMI models.

## 4 Discussion

Partially consistent with our hypothesis, the present results show that specific dimensions of perceived stress are associated with reward-driven eating. In the primary analysis sample overall perceived stress was not independently associated with any reward-driven eating outcome after correction for multiple testing. In contrast, Helplessness showed consistent independent associations with reward-driven eating behavior in models in which both PSS Helplessness and PSS Self-Efficacy were entered simultaneously. Contrary to our hypothesis Self-Efficacy was not independently associated with any reward-driven eating outcome in these models. These associations of Helplessness were robust to additional adjustment for the lifestyle factors current smoking, dieting, and physical activity.

This study extends prior work using global stress measures by examining specific dimensions of perceived stress and demonstrates that subjective experiences of uncontrollability, as captured by the Helplessness subscale, were the stress dimension most consistently associated with reward-driven eating. Overall, moderation analyses did not provide evidence that BMI or BMI-defined weight status significantly modified stress–reward-driven eating associations.

### 4.1 Association of perceived stress and reward-driven eating behavior with BMI

In the present study, perceived stress was not associated with BMI in the primary analysis sample or in either individuals with normal weight or with obesity. Although prior research has yielded mixed findings, the current result accords with evidence suggesting that perceived stress may not influence body weight directly but may instead be related to BMI indirectly via eating-related processes (Singh et al., 2024; Shimanoe et al., 2015; Tenk et al., 2018; Cuevas et al., 2021; Kumar et al., 2022; Dakanalis et al., 2024). Moreover, longitudinal evidence indicates that only prolonged stress predicts subsequent BMI increase (Wardle et al., 2011; Bamert et al., 2025). In the primary analysis sample, BMI was positively associated with PFS Availability, PFS Present, trait food craving, and uncontrolled eating. However, none of these reward-driven eating measures was significantly associated with BMI within the normal-weight or obesity subgroup. This may partly reflect limited statistical power and potentially the relatively low sensitivity of BMI as an index of obesity (Sweatt et al., 2024). Taken together, these findings support the notion that perceived stress may be more closely associated with eating behavior than with BMI (Tuluhong and Han, 2023; Sinha and Jastreboff, 2013; Adam and Epel, 2007; Kumar et al., 2022).

Although the present findings did not support a direct association between perceived stress and BMI, BMI may nevertheless be associated with differences in the associations between perceived stress and reward-driven eating. This possibility was explored in the moderation analyses in the present study.

Across the primary analysis sample and both BMI-defined subgroups, PSS Total was strongly positively associated with PSS Helplessness and strongly negatively associated with PSS Self-Efficacy, while the two subscales were negatively correlated. This pattern was consistent across groups, although the inverse association between the subscales was not significant in the obesity subgroup, likely due to its smaller sample size. This difference may reflect variation in the strength of the association across weight groups, although the underlying reasons remain unclear and further studies in larger samples are needed to determine whether these patterns reflect meaningful differences or sample-specific variation. Overall, these findings support the construct validity of the PSS and its established two-factor structure (Harris et al., 2023; Klein et al., 2016; Cohen et al., 1983).

### 4.2 Perceived stress dimensions and reward-driven eating

Partially consistent with our hypothesis, the present findings demonstrate associations between perceived stress and reward-driven eating behavior, but these associations depended on the dimension of perceived stress examined. In the primary analysis sample, PSS Total was not independently associated with any reward-driven eating outcome after FDR correction. Importantly, in models in which PSS Helplessness and PSS Self-Efficacy were entered simultaneously, Helplessness showed consistent independent associations with several reward-driven eating outcomes after accounting for shared variance between PSS subscales and adjustment for the relevant covariates. Although no causal relationship can be drawn based on the cross-sectional nature of this study, results may indicate that stress-related perceived uncontrollability may be more consistently related to reward-driven eating than perceived coping capacity.

Specifically, higher Helplessness was independently associated with higher hedonic hunger, as reflected by higher PFS Total scores and with higher PFS Availability scores. Associations with PFS Present and PFS Taste did not remain significant after FDR correction. This suggests that the experience of uncontrollability is associated with heightened awareness of and motivation for palatable foods. Moreover, the results suggest that higher perceived stress, and again particularly stress-related perceived uncontrollability, was associated with greater responsiveness to food cues specifically when food is readily available, but not when it is merely present or has been tasted. This finding may be particularly relevant in Westernized, food-abundant countries characterized by constant food availability. Potential underlying mechanisms require further exploration in future longitudinal studies.

In contrast, Self-Efficacy was not independently associated with any measure of reward-driven eating behavior, in models in which both PSS subscales were entered simultaneously. Although higher stress-related Self-Efficacy has previously been linked to more adaptive coping and self-regulation, the present findings may indicate that these strategies are of less relevance in the context of reward-driven eating (Harris et al., 2023; Klein et al., 2016; Cohen et al., 1983).

This finding nevertheless underscores the importance of distinguishing between dimensions of perceived stress rather than relying solely on the overall PSS score. The absence of FDR-significant independent associations for PSS Total in the primary analysis sample, contrasted with the consistent associations observed for Helplessness, further supporting the relevance of examining specific dimensions of perceived stress. Although most previous research has rarely examined these dimensions separately, the existing literature nonetheless supports a general positive association between perceived stress and hedonic hunger (Tomiyama et al., 2011; Tuluhong and Han, 2023; Adam and Epel, 2007).

In addition, the present findings indicate that Helplessness demonstrated independent associations with higher levels of trait food craving and uncontrolled eating, whereas Self-Efficacy showed no independent associations in models in which both PSS subscales were entered simultaneously. Positive associations between perceived stress and both trait food craving and uncontrolled eating are broadly supported by previous research (Jastreboff et al., 2013; Sinha and Jastreboff, 2013; Adam and Epel, 2007; Kumar et al., 2022; Järvelä-Reijonen et al., 2016). The current results extend this literature by demonstrating that, after accounting for the overlap between the two dimensions of perceived stress, perceived uncontrollability was more consistently associated with these facets of reward-driven eating behaviors than perceived coping capacity.

Uncontrolled eating is thought to reflect reduced self-regulatory control in the context of heightened reward responsiveness, a pattern that may be particularly relevant among individuals experiencing greater perceived uncontrollability during stressful situations (Leehr et al., 2015; Sinha and Jastreboff, 2013; Adam and Epel, 2007). The present findings further suggest that particularly stress-related perceived uncontrollability, may be associated with *dispositional tendencies* in the context of reward-driven eating. Thus, interventions targeting subjective perceived uncontrollability in stress experiences may have the potential to contribute to enduring changes in eating behavior.

The present findings are broadly consistent with contemporary psychobiological models of stress-related eating, which propose that particularly experiences of uncontrollability during stress are accompanied by enhanced reward responsiveness and diminished inhibitory control, thereby increasing the motivation for palatable foods (Tomiyama et al., 2011; Sinha and Jastreboff, 2013; Adam and Epel, 2007). However, the present study cannot test underlying psychobiological mechanisms; therefore, possible explanations by these models why Helplessness was independently associated with reward-driven eating and moreover Self-Efficacy wasn’t in simultaneous models, remain speculative. Furthermore, adjustment for current hunger did not eliminate the central Helplessness associations, suggesting that these associations were not solely attributable to differences in acute hunger. The overall pattern of associations was also preserved in sensitivity analyses additionally adjusting for smoking, physical activity, or current dieting, supporting the robustness of the central Helplessness findings to these lifestyle-related factors.

Taken together, the results emphasize the importance of differentiating between distinct stress dimensions, to identify individuals at risk for reward-driven eating and suggest that perceived uncontrollability represents the dimension of perceived stress most consistently associated with reward-driven eating.

### 4.3 BMI, weight status, and stress–reward-driven eating associations

Descriptively, associations between perceived stress and reward-driven eating appeared more consistent among participants with obesity than among participants with normal weight. Within the obesity subgroup, higher perceived stress levels, particularly higher Helplessness scores, were independently associated with greater hedonic hunger, trait food craving and uncontrolled eating. In contrast, no independent association remained significant after FDR correction in the normal-weight subgroup.

As in the primary analysis sample, perceived uncontrollability during stressful experiences, as assessed by the Helplessness subscale, emerged as the stress dimension most consistently associated with reward-driven eating within the subgroup with obesity, whereas Self-Efficacy was not independently associated with any reward-driven eating outcome in models in which both PSS subscales were entered simultaneously, in any subgroup.

The descriptive subgroup pattern is consistent with previous studies reporting stronger associations between stress-related processes and reward-driven eating among individuals with obesity (Jastreboff et al., 2013; Sinha and Jastreboff, 2013; Tryon et al., 2013; Hill et al., 2022). Although exploratory in nature, the observed pattern is also consistent with neurocognitive models proposing that obesity may be characterized by alterations in reward responsiveness and self-regulatory processes that could increase susceptibility to stress-induced increases in hedonic hunger, food craving and uncontrolled eating (Adam and Epel, 2007; Kumar et al., 2022). However, the present subgroup findings alone cannot establish that these associations are stronger in obesity.

These mechanisms were not directly assessed in the present study and therefore remain speculative explanations for the observed associations. Furthermore, the present findings should be interpreted cautiously. Thus, although the subgroup analyses may indicate a pattern worthy of further investigation, the present data do not provide robust statistical support for the hypothesis that BMI or BMI-defined weight status modifies the association between perceived stress and reward-driven eating. While several interaction effects reached nominal significance, none remained significant after correction for multiple testing. Thus, also the subgroup analyses may indicate a pattern worthy of further investigation, the present data do not provide robust statistical support for the hypothesis that BMI or obesity modifies the association between perceived stress and reward-driven eating.

Notably, participants with obesity and participants with normal weight showed substantial overlap across BMI-defined weight groups. In the primary comparison between participants with normal weight and obesity, none of the perceived stress or reward-driven eating variables differed significantly after FDR correction. This finding is consistent with evidence suggesting substantial heterogeneity and overlap in stress- and reward-related eating traits across BMI-defined weight groups, indicating that these psychological constructs are not uniformly elevated in obesity (Espel-Huynh et al., 2018; Crane et al., 2023; Harris et al., 2023; Grol et al., 2022). However, the modest subgroup sizes, particularly the obesity subgroup, limit the precision of these comparisons, and the absence of statistically significant differences should not be interpreted as evidence of equivalence between weight groups, particularly as some previous studies have reported differences across weight status (Jastreboff et al., 2013; Sinha and Jastreboff, 2013; Lowe et al., 2009; Löffler et al., 2015). The primary analytical framework compared participants with normal weight (*n* = 47) and obesity (*n* = 25), while excluding participants with overweight (*n* = 18) from primary inferential analyses. This approach enabled a more distinct comparison of the two BMI-defined groups while avoiding assignment of participants with overweight to either primary group. Nevertheless, BMI remains an indirect measure of adiposity, and larger studies incorporating direct or complementary measures of body composition are needed to determine whether adiposity modifies stress–reward-driven eating associations.

Taken together, the results suggest that stress-related Helplessness is associated with reward-driven eating in the primary analysis sample and shows particularly consistent associations within the obesity subgroup. Although descriptive subgroup analyses indicated more consistent associations among participants with obesity, the absence of FDR-significant interaction effects does not support the conclusion that these associations differ systematically according to BMI or BMI-defined weight status. Larger studies incorporating direct or complementary measures of adiposity are required or complementary measures of adiposity to determine whether adiposity meaningfully modifies these relationships.

### 4.4 Strengths and limitations

A major strength of this study is that the two dimensions of perceived stress were examined simultaneously within the same regression models, allowing the independent associations of Helplessness and Self-Efficacy with reward-driven eating to be estimated after accounting for their shared variance. In addition, multiple facets of reward-driven eating were assessed, providing a comprehensive perspective. Primary inferential analyses directly compared participants with normal weight and obesity, while moderation analyses formally examined whether BMI or BMI-defined weight status modified associations between perceived stress and reward-driven eating. This approach avoided combining participants with overweight with either of the primary comparison groups. However, the moderation analyses did not provide robust evidence of effect modification by BMI or weight status after correction for multiple testing.

Several limitations should be acknowledged. First, the cross-sectional design precludes causal conclusions. Therefore, longitudinal and experimental studies are needed. Second, the reliance on self-report measures introduces the potential for social desirability and awareness biases and may not fully reflect behavioral or physiological manifestations of stress and eating behavior. Third, the BMI-defined subgroups, particularly the subgroup with obesity were relatively small, which may have reduced statistical power and limited sensitivity for subgroup comparisons and moderation analyses.

Fourth, participants with overweight (*n* = 18) were excluded from the primary inferential analyses, resulting in a primary analysis sample of *N* = 72 comprising participants with normal weight (*n* = 47) and obesity (*n* = 25). Although this provided a more distinct comparison between the two BMI-defined groups and avoided assigning participants with overweight to either primary group, it reduced the effective sample size and limits the extent to which the primary findings can be generalized to individuals with overweight. The overweight subgroup was retained descriptively, but its relatively small size precluded adequately powered separate inferential analyses. Accordingly, the present study cannot determine whether stress–reward-driven eating associations among individuals with overweight resemble those observed in participants with normal weight, those with obesity, or represent a distinct pattern. Future studies with larger, prospectively defined samples should examine participants with normal weight, overweight, and obesity separately. *Post hoc* sensitivity analyses indicated limited power for smaller subgroup and interaction effects, particularly in the obesity subgroup, which are common in psychosocial moderation models.

Therefore, non-significant findings, particularly regarding effect modification by weight status and BMI, should be interpreted cautiously, and replication in larger samples is warranted. Further, BMI was used to define weight-status groups but does not directly measure adiposity or body-fat distribution (Sweatt et al., 2024), and complementary anthropometric or body-composition measures were not incorporated into the present analyses. Moreover, the recently proposed distinction between preclinical and clinical obesity could not be assessed, as obesity-related organ dysfunction and functional impairment were not systematically considered in these analyses (Rubino et al., 2025).

Fifth, the predominance of female participants may limit generalizability. Although the primary regression analyses were adjusted for age, sex, BMI, and current hunger, and sensitivity analyses additionally adjusted for the lifestyle variables dieting status, smoking, and physical activity, we did not evaluate several potentially relevant factors such as depressive symptoms, anxiety, sleep quality, socioeconomic status, medication use, or hormonal status. The robustness of the central Helplessness associations to the three additional sensitivity adjustments reduces concern that these findings were attributable to these measured lifestyle-related factors, but residual and unmeasured confounding cannot be excluded.

## 5 Conclusion

In conclusion, this study extends prior research by demonstrating that, after accounting for the overlap between the two dimensions of perceived stress, Helplessness, but not Self-Efficacy, was independently associated with multiple facets of reward-driven eating. Importantly, overall perceived stress as assessed by PSS Total was not independently associated with any reward-driven eating outcome after correction for multiple testing in the primary analysis sample, whereas Helplessness remained independently associated with PFS Total, PFS Availability, trait food craving, and uncontrolled eating. These central Helplessness associations were robust to additional adjustment for current smoking, physical activity, and current dieting. These findings highlight that uncontrollability may represent the dimension of perceived stress most consistently related to reward-driven eating and underscore the importance of distinguishing between distinct dimensions of perceived stress rather than relying solely on the overall PSS score. Although descriptive subgroup analyses suggested more consistent associations in participants with obesity than in participants with normal weight, these observations were not supported by the formal moderation analyses after correction for multiple testing. Perceived uncontrollability may nevertheless represent a potential target for interventions aimed at reducing reward-driven eating behavior. Future studies with larger samples are warranted to determine whether BMI or BMI-defined weight status meaningfully modifies the relationship between perceived stress and reward-driven eating.

## Data Availability

The raw data supporting the conclusions of this article will be made available by the authors, without undue reservation.
