# Supplementary material for: Stress-related helplessness is independently associated with reward-driven eating in adults with BMI-defined normal weight and obesity
Source: Front Psychol. 2026 Oct 14;17:1820806. doi: 10.3389/fpsyg.2026.1820806 (PMC13713407; doi:10.3389/fpsyg.2026.1820806)
Supplement: Supplementary file 1 [file Table_1.DOCX]

**Supplements**

**Supplement Table 1: Association of perceived stress and outcomes with potential confounding variables in primary analysis sample.**

| N = 72 | BMI | Age | Sex | Current hunger | Current dieting | Current smoking | Physical activity |
| --- | --- | --- | --- | --- | --- | --- | --- |
| PSS Total | r = 0.034 p = 0.775 | r = -0.137 p = 0.252 | r = -0.147 p = 0.217 | **r = 0.305 p = 0.009** | r = -0.094 p = 0.433 | r = -0.062 p = 0.606 | r = -0.026 p = 0.837 |
| PSS Helplessness | r = -0.102 p = 0.396 | r = -0.212 p = 0.074 | r = -0.063 p = 0.597 | **r = 0.238 p = 0.044** | r = -0.080 p = 0.502 | r = -0.148 p = 0.213 | r = 0.023 p = 0.855 |
| PSS Self-Efficacy | r = -0.180 p = 0.131 | r = -0.014 p = 0.909 | r = 0.172 p = 0.149 | r = -0.217 p = 0.067 | r = 0.068 p = 0.569 | r = -0.052 p = 0.661 | r = 0.066 p = 0.599 |
| PFS Total | r = 0.195 p = 0.102 | r = -0.155 p = 0.196 | r = -0.182 p = 0.128 | r = 0.059 p = 0.624 | r = -0.020 p = 0.871 | r = 0.088 p = 0.467 | r = 0.005 p = 0.966 |
| PFS Availability | **r = 0.286 p = 0.016** | r = -0.127 p = 0.290 | **r = -0.255 p = 0.032** | r = 0.018 p = 0.882 | r = 0.037 p = 0.759 | r = 0.029 p = 0.811 | r = -0.080 p = 0.526 |
| PFS Present | **r = 0.272 p = 0.021** | r = -0.098 p = 0.414 | r = -0.153 p = 0.200 | r = 0.089 p = 0.458 | r = 0.083 p = 0.489 | r = -0.016 p = 0.894 | r = 0.035 p = 0.778 |
| PFS Taste | r = -0.069 p = 0.564 | r = -0.230 p = 0.051 | r = -0.079 p = 0.509 | r = -0.004 p = 0.973 | r = -0.055 p = 0.643 | r = 0.018 p = 0.880 | r = 0.091 p = 0.468 |
| FC | **r = 0.266 p = 0.031** | r = -0.058 p = 0.645 | **r = -0.317 p = 0.010** | r = -0.011 p = 0.930 | **r = 0.266 p = 0.031** | r = 0.082 p = 0.514 | r = -0.028 p = 0.834 |
| UE | **r = 0.310 p = 0.010** | r = 0.116 p = 0.346 | r = -0.108 p = 0.383 | r = -0.149 p = 0.226 | **r = 0.279 p = 0.021** | r = 0.031 p = 0.805 | r = -0.118 p = 0.360 |

Supplement Table 1: Spearman´s rank correlation coefficient was used to assess associations between perceived stress and potential covariates in the primary analysis sample comprising participants with normal weight and obesity. Results are presented as correlation coefficient (r) with corresponding p-values. A p-value < 0.05 was considered statistically significant; statistically significant results are highlighted in bold. Trait food craving was assessed with the Food Craving Questionnaire- Trait reduced, trait uncontrolled eating was assessed with the Three-Factor Eating Questionnaire. Smoking was defined as smoking at least one cigarette per day. Current hunger was assessed on a 100 mm visual analogue scale (0 = not hungry; 100 mm = very hungry). Physical activity was assessed using the International Physical Activity Questionnaire. Current dieting was assessed as a dichotomous variable (yes/no). Abbreviations: N, number. PSS, Perceived Stress Scale, FC, trait Food craving, UE, trait uncontrolled eating. Physical activity was missing for six participants with normal weight. Trait food craving was missing for one participant with normal weight and five participants with obesity; uncontrolled eating was missing for four participants with obesity;

**Supplement Table 2:** **Association of perceived stress and outcomes with potential confounding variables in individuals with obesity.**

| N = 25 | BMI | Age | Sex | Current hunger | Current dieting | Current  smoking | Physical activity |
| --- | --- | --- | --- | --- | --- | --- | --- |
| PSS Total | r = 0.119,  p = 0.572 | r = -0.362,  p = 0.075 | r = -0.179,  p = 0.391 | **r = 0.555,**  **p = 0.004** | r = -0.153,  p = 0.464 | r = -0.145,  p = 0.490 | r = -0.117,  p = 0.578 |
| PSS Helplessness | r = -0.153,  p = 0.466 | r = -0.133,  p = 0.527 | r = -0.186,  p = 0.373 | r = 0.227,  p = 0.275 | r = -0.098,  p = 0.641 | r = -0.336,  p = 0.100 | r = -0.106,  p = 0.615 |
| PSS Self-Efficacy | r = -0.108,  p = 0.607 | **r = 0.403,**  **p = 0.046** | r = 0.193,  p = 0.356 | **r = -0.643, p < 0.001** | r = -0.168,  p = 0.421 | r = -0.092,  p = 0.662 | r = 0.032,  p = 0.878 |
| PFS Total | r = -0.057,  p = 0.787 | r = -0.178,  p = 0.394 | r = -0.351,  p = 0.085 | r = 0.121,  p = 0.563 | r = -0.298,  p = 0.147 | r = -0.136,  p = 0.516 | r = -0.200,  p = 0.339 |
| PFS Availability | r = 0.099,  p = 0.639 | r = -0.392,  p = 0.053 | r = -0.244,  p = 0.239 | r = 0.218,  p = 0.295 | r = -0.222,  p = 0.285 | r = -0.083,  p = 0.692 | r= -0.236,  p = 0.256 |
| PFS Present | r = -0.025,  p = 0.905 | r = -0.202,  p = 0.332 | **r = -0.407,**  **p = 0.044** | r = 0.134,  p = 0.524 | r = -0.237,  p = 0.254 | r = -0.343,  p = 0.094 | r = -0.131,  p = 0.532 |
| PFS Taste | r = -0.366,  p = 0.072 | r = 0.239,  p = 0.250 | r = -0.269,  p = 0.194 | r = -0.027,  p = 0.899 | r = -0.139,  p = 0.507 | r = -0.053,  p = 0.801 | r = -0.076,  p = 0.719 |
| FC | r = -0.095,  p = 0.691 | r = -0.092,  p = 0.700 | **r = -0.637,**  **p = 0.003** | r = 0.079,  p = 0.740 | r = -0.295,  p = 0.234 | r = 0.266,  p = 0.257 | r = -0.067,  p = 0.778 |
| UE | r = 0.042,  p = 0.855 | r = 0.308,  p = 0.174 | r = -0.117,  p = 0.612 | r = -0.195,  r = 0.397 | r = 0.140,  p = 0.555 | r = -0.314,  p = 0.177 | r = -0.176,  p = 0.445 |

Supplement Table 2: Spearman´s rank correlation coefficient was used to assess associations between perceived stress and potential confounding variables in the subsample with obesity. Results are presented as correlation coefficient (r) with corresponding p-values. A p-value < 0.05 was considered statistically significant; statistically significant results are highlighted in bold. Trait food craving was assessed with the Food Craving Questionnaire- Trait reduced, trait uncontrolled eating was assessed with the Three-Factor Eating Questionnaire. Smoking was defined as smoking at least one cigarette per day. Current hunger was assessed on a 100 mm visual analogue scale (0 = not hungry; 100 mm = very hungry). Physical activity was assessed using the International Physical Activity Questionnaire. Current dieting was assessed as a dichotomous variable (yes/no). Abbreviations: N, number. PSS, Perceived Stress Scale, FC, trait food craving, UE, trait uncontrolled eating. Data were missing for trait food craving in 5 individuals, for trait uncontrolled eating in 4 individuals.

**Supplement Table 3: Association of perceived stress and potential confounding variables in individuals with normal weight.**

| N = 47 | BMI | Age | Sex | Current hunger | Current dieting | Current  smoking | Physical activity |
| --- | --- | --- | --- | --- | --- | --- | --- |
| PSS Total | r = -0.099 p = 0.506 | r = -0.107 p = 0.473 | r = -0.103 p = 0.492 | r = 0.243 p = 0.100 | r = -0.117 p = 0.434 | r = -0.042 p = 0.778 | r = 0.155 p = 0.334 |
| PSS Helplessness | r = -0.038 p = 0.800 | r = -0.194 p = 0.191 | r = 0.015 p = 0.919 | r = 0.256 p = 0.083 | r = -0.066 p = 0.658 | r = -0.037 p = 0.807 | r = 0.195 p = 0.222 |
| PSS Self efficacy | r = 0.115 p = 0.442 | r = -0.023 p = 0.877 | r = 0.151 p = 0.311 | r = -0.191 p = 0.199 | r = 0.149 p = 0.318 | r = 0.065 p = 0.663 | r = -0.048 p = 0.766 |
| PFS Total | r = 0.191 p = 0.204 | r = -0.247 p = 0.098 | r = -0.080 p = 0.596 | r = 0.043 p = 0.778 | r = 0.145 p = 0.337 | r = 0.242 p = 0.105 | r = 0.284 p = 0.076 |
| PFS Availability | r = 0.271 p = 0.068 | r = -0.119 p = 0.429 | r = -0.281 p = 0.058 | r = -0.096 p = 0.524 | r = 0.162 p = 0.283 | r = 0.097 p = 0.522 | r = 0.186 p = 0.249 |
| PFS Present | r = 0.113 p = 0.448 | r = -0.226 p = 0.126 | r = -0.086 p = 0.563 | r = 0.137 p = 0.357 | r = 0.145 p = 0.331 | r = 0.108 p = 0.471 | r = 0.291 p = 0.065 |
| PFS Taste | r = 0.053 p = 0.722 | **r = -0.403 p = 0.005** | r = 0.024 p = 0.874 | r = -0.042 p = 0.780 | r = 0.055 p = 0.715 | r = 0.088 p = 0.558 | r = 0.243 p = 0.126 |
| FCQ | r = -0.099 p = 0.506 | r = -0.107 p = 0.473 | r = -0.103 p = 0.492 | r = 0.243 p = 0.100 | r = -0.117 p = 0.434 | r = -0.042 p = 0.778 | r = 0.155 p = 0.334 |
| UE | r = -0.038 p = 0.800 | r = -0.194 p = 0.191 | r = 0.015 p = 0.919 | r = 0.256 p = 0.083 | r = -0.066 p = 0.658 | r = -0.037 p = 0.807 | r = 0.195 p = 0.222 |

Supplement Table 3: Spearman´s rank correlation coefficient was used to assess associations between perceived stress and potential covariates in normal weight subsample (BMI 18.5–24.9 kg/m²). Results are presented as correlation coefficient (r) with corresponding p-values. A p-value < 0.05 was considered statistically significant; statistically significant results are highlighted in bold. Trait food craving was assessed with the Food Craving Questionnaire- Trait reduced, trait uncontrolled eating was assessed with the Three-Factor Eating Questionnaire. Smoking was defined as smoking at least one cigarette per day. Current hunger was assessed on a 100 mm visual analogue scale (0 = not hungry; 100 mm = very hungry). Current dieting was assessed as a dichotomous variable (yes/no). Physical activity was assessed using the International Physical Activity Questionnaire. Abbreviations: N, number. PSS, Perceived Stress Scale, FC, trait food craving, UC, trait uncontrolled eating, PFS, Power of Food Scale. Trait food craving and PFS Total/PFS Availability were each missing for one participant; physical activity was missing for six participants.

**Supplement Table 4: Moderation effects of weight status (normal weight vs obesity) on the association between perceived stress and reward-driven eating outcomes.**

| Predictor | Outcome | b | SE | 95%CI | ΔR² | Raw p /q FDR |
| --- | --- | --- | --- | --- | --- | --- |
| PSS HL | FC | 1.050 | 0.897 | -0.746; 2.847 | 0.018 | 0.247 / 0.444 |
|  | PFS Total | 0.045 | 0.048 | -0.051; 0.140 | 0.013 | 0.354 / 0.510 |
|  | PFS Availability | 0.082 | 0.043 | -0.004; 0.169 | 0.032 | 0.062 / 0.231 |
|  | PFS Present | -0.009 | 0.083 | -0.175; 0.156 | 0.000 | 0.908 / 0.908 |
|  | PFS Taste | 0.040 | 0.050 | -0.059; 0.139 | 0.008 | 0.423 / 0.544 |
|  | UE | 0.196 | 0.203 | -0.210; 0.601 | 0.015 | 0.339 / 0.509 |
| PSS Total | FC | 1.039 | 0.760 | -0.480; 2.559 | 0.036 | 0.176 / 0.353 |
|  | PFS Total | 0.069 | 0.041 | -0.013; 0.151 | 0.060 | 0.096 / 0.231 |
|  | PFS Availability | 0.092 | 0.033 | 0.027; 0.157 | 0.078 | 0.006 / 0.112 |
|  | PFS Present | 0.026 | 0.069 | -0.112; 0.164 | 0.004 | 0.705 / 0.746 |
|  | PFS Taste | 0.070 | 0.041 | -0.012; 0.152 | 0.046 | 0.094 / 0.231 |
|  | UE | 0.197 | 0.141 | -0.085; 0.478 | 0.029 | 0.167 / 0.353 |
| PSS SE | FC | -1.093 | 1.480 | -4.055; 1.870 | 0.009 | 0.463 / 0.556 |
|  | PFS Total | -0.160 | 0.089 | -0.339; 0.019 | 0.072 | 0.079 / 0.231 |
|  | PFS Availability | -0.188 | 0.085 | -0.358; -0.018 | 0.072 | 0.031 / 0.173 |
|  | PFS Present | -0.087 | 0.146 | -0.378; 0.204 | 0.011 | 0.553 / 0.642 |
|  | PFS Taste | -0.166 | 0.088 | -0.343; 0.010 | 0.059 | 0.064 / 0.231 |
|  | UE | -0.217 | 0.288 | -0.793; 0.359 | 0.008 | 0.455 / 0.556 |

### Supplement Table 4: Values represent interaction terms from PROCESS Model 1 analyses in the primary analysis sample comprising participants with normal weight and obesity. BMI-defined weight status was entered as the categorical moderator. Models were adjusted for age, sex, and current hunger. Models including **PSS Helplessness** or PSS Self-Efficacy as the focal predictor were additionally adjusted for the other **PSS subscale**. After FDR correction, none of the interaction effects remained statistically significant. Abbreviation: FC, trait food cravings; UE, trait uncontrolled eating; PFS, Power of Food Scale, PSS; Perceived Stress Scale; HL, Helplessness; SE, Self-Efficacy; b, unstandardized regression coefficient; SE, standard error; ΔR², change in variance explained by the interaction term; 95% CI, 95% confidence interval.

### Supplement Table 5: Moderation effects of continous BMI on the association between perceived stress and eating related outcomes.

| Predictor | Outcome | b | SE | 95%CI | ΔR² | Raw p/qFDR |
| --- | --- | --- | --- | --- | --- | --- |
| PSS HL | FC | 0.063 | 0.051 | -0.039; 0.164 | 0.021 | 0.222 / 0.420 |
|  | PFS Total | 0.004 | 0.004 | -0.004; 0.012 | 0.031 | 0.338 / 0.509 |
|  | PFS Availability | 0.006 | 0.004 | -0.002; 0.013 | 0.044 | 0.154 / 0.347 |
|  | PFS Present | 0.003 | 0.006 | -0.009; 0.014 | 0.007 | 0.647 / 0.706 |
|  | PFS Taste | 0.003 | 0.004 | -0.004; 0.011 | 0.014 | 0.415 / 0.544 |
|  | UE | 0.020 | 0.011 | -0.002; 0.043 | 0.049 | 0.078 / 0.231 |
| PSS Total | FC | 0.041 | 0.040 | -0.039; 0.120 | 0.017 | 0.309 / 0.509 |
|  | PFS Total | 0.005 | 0.002 | 0.001; 0.009 | 0.087 | 0.031 / 0.173 |
|  | PFS Availability | 0.006 | 0.002 | 0.002; 0.009 | 0.093 | 0.003 / 0.097 |
|  | PFS Present | 0.003 | 0.004 | -0.004; 0.011 | 0.022 | 0.380 / 0.526 |
|  | PFS Taste | 0.004 | 0.002 | 0.001; 0.008 | 0.056 | 0.028 / 0.173 |
|  | UE | 0.014 | 0.008 | -0.002; 0.030 | 0.046 | 0.093 / 0.231 |
| PSS SE | FC | -0.009 | 0.070 | -0.150; 0.132 | 0.000 | 0.898 / 0.908 |
|  | PFS Total | -0.009 | 0.004 | -0.018; -0.000 | 0.085 | 0.046 / 0.208 |
|  | PFS Availability | -0.010 | 0.004 | -0.018; -0.001 | 0.074 | 0.031 / 0.173 |
|  | PFS Present | -0.006 | 0.006 | -0.019; 0.007 | 0.021 | 0.334 / 0.509 |
|  | PFS Taste | -0.009 | 0.004 | -0.018; -0.001 | 0.070 | 0.034 / 0.173 |
|  | UE | -0.009 | 0.019 | -0.047; 0.029 | 0.005 | 0.642 / 0.706 |

### Supplement Table 5: Values represent interaction terms from PROCESS Model 1 analyses in the primary analysis sample comprising participants with normal weight and obesity. BMI was entered as a continuous moderator. Models were adjusted for age, sex, and current hunger. Models including **PSS Helplessness** or PSS Self-Efficacy as the focal predictor were additionally adjusted for the other **PSS subscale**. After FDR-corrected analyses, none of the interaction effects remained statistically significant. Abbreviation: FC, trait food cravings ; UE, trait uncontrolled eating; PFS, Power of Food Scale, PSS; Perceived Stress Scale; HL, Helplessness; SE, Self-Efficacy; b, unstandardized regression coefficient; SE, standard error; ΔR², change in variance explained by the interaction term; 95% CI, 95% confidence interval.

### Supplement Table 6: Sensitivity analyses with current smoking as an additional covariate.

| **Outcome** | **Predictor** | **N** | **B (95% CI)** | **β** | **Raw p** |
| --- | --- | --- | --- | --- | --- |
| **Primary analysis sample** | | | | | |
| PFS Total | PSS Total | 71 | 0.022 (-0.009; 0.053) | 0.174 | 0.167 |
|  | Helplessness | 71 | 0.069 (0.022; 0.115) | 0.374 | 0.004 |
|  | Self-Efficacy | 71 | 0.053 (-0.011; 0.117) | 0.220 | 0.105 |
| PFS Availability | PSS Total | 71 | 0.039 (0.004; 0.074) | 0.263 | 0.028 |
|  | Helplessness | 71 | 0.079 (0.027; 0.132) | 0.367 | 0.004 |
|  | Self-Efficacy | 71 | 0.026 (-0.047; 0.098) | 0.090 | 0.484 |
| PFS Present | PSS Total | 72 | 0.020 (-0.025; 0.066) | 0.115 | 0.369 |
|  | Helplessness | 72 | 0.070 (0.002; 0.138) | 0.270 | 0.044 |
|  | Self-Efficacy | 72 | 0.059 (-0.035; 0.153) | 0.173 | 0.215 |
| PFS Taste | PSS Total | 72 | 0.004 (-0.034; 0.041) | 0.026 | 0.843 |
|  | Helplessness | 72 | 0.049 (-0.007; 0.106) | 0.231 | 0.086 |
|  | Self-Efficacy | 72 | 0.069 (-0.009; 0.147) | 0.248 | 0.081 |
| Uncontrolled eating | PSS Total | 68 | 0.132 (0.001; 0.263) | 0.256 | 0.048 |
|  | Helplessness | 68 | 0.338 (0.146; 0.530) | 0.451 | < 0.001 |
|  | Self-Efficacy | 68 | 0.194 (-0.069; 0.456) | 0.196 | 0.145 |
| Trait food craving | PSS Total | 66 | 0.407 (-0.206; 1.020) | 0.167 | 0.189 |
|  | Helplessness | 66 | 1.480 (0.560; 2.400) | 0.410 | 0.002 |
|  | Self-Efficacy | 66 | 1.205 (-0.018; 2.427) | 0.260 | 0.053 |
| **Obesity** | | | | | |
| PFS Total | PSS Total | 25 | 0.080 (0.018; 0.142) | 0.596 | 0.014 |
|  | Helplessness | 25 | 0.078 (0.005; 0.152) | 0.446 | 0.037 |
|  | Self-Efficacy | 25 | -0.084 (-0.223; 0.054) | -0.331 | 0.217 |
| PFS Availability | PSS Total | 25 | 0.114 (0.045; 0.183) | 0.685 | 0.003 |
|  | Helplessness | 25 | 0.113 (0.032; 0.195) | 0.519 | 0.009 |
|  | Self-Efficacy | 25 | -0.116 (-0.270; 0.039) | -0.365 | 0.135 |
| PFS Present | PSS Total | 25 | 0.019 (-0.063; 0.102) | 0.118 | 0.627 |
|  | Helplessness | 25 | 0.024 (-0.073; 0.122) | 0.112 | 0.606 |
|  | Self-Efficacy | 25 | -0.003 (-0.188; 0.182) | -0.009 | 0.976 |
| PFS Taste | PSS Total | 25 | 0.087 (0.019; 0.156) | 0.602 | 0.016 |
|  | Helplessness | 25 | 0.080 (-0.001; 0.161) | 0.420 | 0.053 |
|  | Self-Efficacy | 25 | -0.112 (-0.266; 0.041) | -0.407 | 0.141 |
| Uncontrolled eating | PSS Total | 21 | 0.395 (0.069; 0.721) | 0.666 | 0.021 |
|  | Helplessness | 21 | 0.509 (0.143; 0.874) | 0.655 | 0.010 |
|  | Self-Efficacy | 21 | -0.007 (-0.696; 0.681) | -0.007 | 0.982 |
| Trait food craving | PSS Total | 20 | 1.195 (-0.045; 2.435) | 0.482 | 0.058 |
|  | Helplessness | 20 | 1.704 (0.369; 3.039) | 0.525 | 0.016 |
|  | Self-Efficacy | 20 | 0.589 (-1.947; 3.124) | 0.126 | 0.624 |
| **Normal weight** | | | | | |
| PFS Total | PSS Total | 46 | -0.001 (-0.037; 0.035) | -0.006 | 0.968 |
|  | Helplessness | 46 | 0.063 (-0.003; 0.128) | 0.335 | 0.060 |
|  | Self-Efficacy | 46 | 0.086 (0.004; 0.168) | 0.351 | 0.041 |
| PFS Availability | PSS Total | 46 | 0.013 (-0.029; 0.056) | 0.099 | 0.526 |
|  | Helplessness | 46 | 0.070 (-0.008; 0.149) | 0.336 | 0.078 |
|  | Self-Efficacy | 46 | 0.063 (-0.035; 0.162) | 0.231 | 0.203 |
| PFS Present | PSS Total | 47 | 0.013 (-0.042; 0.069) | 0.074 | 0.636 |
|  | Helplessness | 47 | 0.084 (-0.020; 0.188) | 0.304 | 0.110 |
|  | Self-Efficacy | 47 | 0.081 (-0.049; 0.211) | 0.227 | 0.213 |
| PFS Taste | PSS Total | 47 | -0.024 (-0.063; 0.015) | -0.164 | 0.224 |
|  | Helplessness | 47 | 0.031 (-0.042; 0.104) | 0.137 | 0.395 |
|  | Self-Efficacy | 47 | 0.097 (0.006; 0.188) | 0.332 | 0.037 |
| Uncontrolled eating | PSS Total | 47 | 0.063 (-0.082; 0.207) | 0.138 | 0.385 |
|  | Helplessness | 47 | 0.231 (-0.040; 0.503) | 0.328 | 0.093 |
|  | Self-Efficacy | 47 | 0.162 (-0.178; 0.502) | 0.177 | 0.341 |
| Trait food craving | PSS Total | 46 | 0.075 (-0.637; 0.786) | 0.032 | 0.833 |
|  | Helplessness | 46 | 0.700 (-0.778; 2.179) | 0.187 | 0.344 |
|  | Self-Efficacy | 46 | 0.693 (-1.049; 2.435) | 0.148 | 0.426 |

### Only coefficients for the focal PSS predictors for the final model including smoking are shown. PSS Total was examined in separate models; Helplessness and Self-Efficacy were entered simultaneously in subscale models. Models in the primary analysis sample were adjusted for age, sex, BMI, current hunger, and current smoking. BMI-defined subgroup models were adjusted for age, sex, current hunger, and current smoking; BMI was omitted because it defined the stratification. Current smoking was defined as smoking at least one cigarette per day and was entered as binary covariate. Abbreviations: B = unstandardized regression coefficient; β = standardized regression coefficient; CI = confidence interval; PFS = Power of Food Scale; PSS = Perceived Stress Scale.

### Supplement Table 7: Sensitivity analyses with physical activity as an additional covariate.

| **Outcome** | **Predictor** | **N** | **B (95% CI)** | **β** | **Raw p** |
| --- | --- | --- | --- | --- | --- |
| **Primary analysis sample** | | | | | |
| PFS Total | PSS Total | 65 | 0.027 (-0.005; 0.060) | 0.220 | 0.100 |
|  | Helplessness | 65 | 0.069 (0.022; 0.117) | 0.386 | 0.005 |
|  | Self-Efficacy | 65 | 0.043 (-0.023; 0.110) | 0.182 | 0.199 |
| PFS Availability | PSS Total | 65 | 0.045 (0.010; 0.081) | 0.317 | 0.014 |
|  | Helplessness | 65 | 0.082 (0.030; 0.134) | 0.397 | 0.002 |
|  | Self-Efficacy | 65 | 0.016 (-0.057; 0.090) | 0.061 | 0.655 |
| PFS Present | PSS Total | 66 | 0.025 (-0.023; 0.074) | 0.140 | 0.300 |
|  | Helplessness | 66 | 0.069 (-0.002; 0.140) | 0.263 | 0.057 |
|  | Self-Efficacy | 66 | 0.048 (-0.052; 0.148) | 0.139 | 0.345 |
| PFS Taste | PSS Total | 66 | 0.009 (-0.030; 0.048) | 0.064 | 0.642 |
|  | Helplessness | 66 | 0.053 (-0.003; 0.109) | 0.256 | 0.064 |
|  | Self-Efficacy | 66 | 0.064 (-0.015; 0.142) | 0.238 | 0.110 |
| Uncontrolled eating | PSS Total | 62 | 0.152 (0.010; 0.294) | 0.289 | 0.037 |
|  | Helplessness | 62 | 0.349 (0.147; 0.552) | 0.460 | 0.001 |
|  | Self-Efficacy | 62 | 0.172 (-0.110; 0.453) | 0.172 | 0.227 |
| Trait food craving | PSS Total | 60 | 0.479 (-0.184; 1.143) | 0.196 | 0.153 |
|  | Helplessness | 60 | 1.556 (0.597; 2.515) | 0.433 | 0.002 |
|  | Self-Efficacy | 60 | 1.176 (-0.108; 2.461) | 0.256 | 0.072 |
| **Obesity** | | | | | |
| PFS Total | PSS Total | 25 | 0.075 (0.014; 0.137) | 0.563 | 0.020 |
|  | Helplessness | 25 | 0.069 (-0.006; 0.143) | 0.390 | 0.068 |
|  | Self-Efficacy | 25 | -0.098 (-0.245; 0.048) | -0.385 | 0.176 |
| PFS Availability | PSS Total | 25 | 0.104 (0.036; 0.172) | 0.626 | 0.005 |
|  | Helplessness | 25 | 0.098 (0.016; 0.180) | 0.447 | 0.022 |
|  | Self-Efficacy | 25 | -0.126 (-0.287; 0.035) | -0.398 | 0.118 |
| PFS Present | PSS Total | 25 | 0.027 (-0.062; 0.115) | 0.160 | 0.536 |
|  | Helplessness | 25 | 0.032 (-0.075; 0.139) | 0.147 | 0.537 |
|  | Self-Efficacy | 25 | -0.008 (-0.219; 0.202) | -0.026 | 0.935 |
| PFS Taste | PSS Total | 25 | 0.080 (0.010; 0.151) | 0.553 | 0.028 |
|  | Helplessness | 25 | 0.064 (-0.020; 0.147) | 0.334 | 0.128 |
|  | Self-Efficacy | 25 | -0.137 (-0.302; 0.028) | -0.497 | 0.098 |
| Uncontrolled eating | PSS Total | 21 | 0.379 (0.033; 0.724) | 0.639 | 0.034 |
|  | Helplessness | 21 | 0.520 (0.133; 0.907) | 0.670 | 0.012 |
|  | Self-Efficacy | 21 | 0.102 (-0.650; 0.853) | 0.091 | 0.775 |
| Trait food craving | PSS Total | 20 | 1.312 (-0.003; 2.627) | 0.530 | 0.050 |
|  | Helplessness | 20 | 1.862 (0.404; 3.320) | 0.574 | 0.017 |
|  | Self-Efficacy | 20 | 0.592 (-2.279; 3.463) | 0.127 | 0.661 |
| **Normal weight** | | | | | |
| PFS Total | PSS Total | 40 | 0.002 (-0.037; 0.040) | 0.014 | 0.932 |
|  | Helplessness | 40 | 0.066 (-0.002; 0.133) | 0.365 | 0.056 |
|  | Self-Efficacy | 40 | 0.084 (0.000; 0.169) | 0.356 | 0.050 |
| PFS Availability | PSS Total | 40 | 0.017 (-0.026; 0.061) | 0.138 | 0.419 |
|  | Helplessness | 40 | 0.073 (-0.005; 0.151) | 0.378 | 0.065 |
|  | Self-Efficacy | 40 | 0.057 (-0.040; 0.155) | 0.225 | 0.241 |
| PFS Present | PSS Total | 41 | 0.014 (-0.048; 0.077) | 0.079 | 0.644 |
|  | Helplessness | 41 | 0.084 (-0.030; 0.198) | 0.299 | 0.144 |
|  | Self-Efficacy | 41 | 0.078 (-0.064; 0.220) | 0.214 | 0.271 |
| PFS Taste | PSS Total | 41 | -0.025 (-0.064; 0.014) | -0.177 | 0.205 |
|  | Helplessness | 41 | 0.038 (-0.031; 0.107) | 0.175 | 0.270 |
|  | Self-Efficacy | 41 | 0.108 (0.023; 0.194) | 0.385 | 0.014 |
| Uncontrolled eating | PSS Total | 41 | 0.081 (-0.083; 0.245) | 0.171 | 0.324 |
|  | Helplessness | 41 | 0.247 (-0.054; 0.547) | 0.341 | 0.104 |
|  | Self-Efficacy | 41 | 0.140 (-0.233; 0.513) | 0.149 | 0.451 |
| Trait food craving | PSS Total | 40 | 0.078 (-0.741; 0.897) | 0.033 | 0.848 |
|  | Helplessness | 40 | 0.905 (-0.731; 2.542) | 0.244 | 0.268 |
|  | Self-Efficacy | 40 | 0.921 (-0.976; 2.817) | 0.199 | 0.331 |

Only coefficients for the focal PSS predictors from the final model including physical activity are shown. PSS Total was examined in separate models; Helplessness and Self-Efficacy were entered simultaneously in subscale models. Physical activity was represented by two dummy variables for low and moderate physical activity, with high physical activity as the reference category. Models in the primary analysis sample were adjusted for age, sex, BMI, current hunger, PA-low, and PA-moderate. Models in the obesity subgroup were adjusted for age, sex, current hunger, PA-low, and PA-moderate. In the normal-weight subgroup, PA-low was omitted because no participant with available physical-activity data was classified as having low physical activity; models therefore included PA-moderate with high physical activity as the reference category. BMI was not included in the BMI-defined subgroup models. Physical-activity data were missing for six participants with normal weight. Analyses used listwise available cases. Raw two-sided p values are reported. B, unstandardized regression coefficient; β, standardized regression coefficient; CI, confidence interval; BMI, body mass index; PA, physical activity; PFS, Power of Food Scale; PSS, Perceived Stress Scale.

**Supplement Table 8: Sensitivity analyses with current dieting as an additional covariate.**

| **Outcome** | **Predictor** | **N** | **B (95% CI)** | **β** | **Raw p** |
| --- | --- | --- | --- | --- | --- |
| **Primary analysis sample** | | | | | |
| PFS Total | PSS Total | 71 | 0.022 (-0.009; 0.053) | 0.176 | 0.161 |
|  | Helplessness | 71 | 0.065 (0.019; 0.111) | 0.353 | 0.007 |
|  | Self-Efficacy | 71 | 0.048 (-0.017; 0.113) | 0.199 | 0.146 |
| PFS Availability | PSS Total | 71 | 0.040 (0.005; 0.074) | 0.270 | 0.024 |
|  | Helplessness | 71 | 0.076 (0.025; 0.128) | 0.354 | 0.004 |
|  | Self-Efficacy | 71 | 0.020 (-0.053; 0.093) | 0.071 | 0.581 |
| PFS Present | PSS Total | 72 | 0.021 (-0.024; 0.066) | 0.119 | 0.352 |
|  | Helplessness | 72 | 0.067 (0.000; 0.135) | 0.258 | 0.052 |
|  | Self-Efficacy | 72 | 0.054 (-0.040; 0.149) | 0.160 | 0.256 |
| PFS Taste | PSS Total | 72 | 0.004 (-0.034; 0.041) | 0.025 | 0.848 |
|  | Helplessness | 72 | 0.047 (-0.009; 0.103) | 0.221 | 0.098 |
|  | Self-Efficacy | 72 | 0.068 (-0.011; 0.146) | 0.243 | 0.091 |
| Uncontrolled eating | PSS Total | 68 | 0.138 (0.012; 0.264) | 0.267 | 0.033 |
|  | Helplessness | 68 | 0.322 (0.136; 0.508) | 0.430 | 0.001 |
|  | Self-Efficacy | 68 | 0.160 (-0.099; 0.419) | 0.162 | 0.223 |
| Trait food craving | PSS Total | 66 | 0.426 (-0.152; 1.004) | 0.174 | 0.146 |
|  | Helplessness | 66 | 1.329 (0.449; 2.210) | 0.369 | 0.004 |
|  | Self-Efficacy | 66 | 0.956 (-0.230; 2.142) | 0.206 | 0.112 |
| **Obesity** | | | | | |
| PFS Total | PSS Total | 25 | 0.082 (0.022; 0.141) | 0.610 | 0.010 |
|  | Helplessness | 25 | 0.082 (0.013; 0.152) | 0.468 | 0.023 |
|  | Self-Efficacy | 25 | -0.079 (-0.218; 0.059) | -0.311 | 0.244 |
| PFS Availability | PSS Total | 25 | 0.114 (0.047; 0.180) | 0.684 | 0.002 |
|  | Helplessness | 25 | 0.115 (0.037; 0.193) | 0.525 | 0.006 |
|  | Self-Efficacy | 25 | -0.110 (-0.265; 0.044) | -0.348 | 0.151 |
| PFS Present | PSS Total | 25 | 0.033 (-0.052; 0.118) | 0.200 | 0.423 |
|  | Helplessness | 25 | 0.044 (-0.055; 0.142) | 0.200 | 0.366 |
|  | Self-Efficacy | 25 | 0.006 (-0.190; 0.201) | 0.018 | 0.951 |
| PFS Taste | PSS Total | 25 | 0.083 (0.015; 0.150) | 0.569 | 0.019 |
|  | Helplessness | 25 | 0.075 (-0.003; 0.153) | 0.394 | 0.060 |
|  | Self-Efficacy | 25 | -0.111 (-0.266; 0.045) | -0.400 | 0.152 |
| Uncontrolled eating | PSS Total | 21 | 0.378 (0.061; 0.695) | 0.637 | 0.023 |
|  | Helplessness | 21 | 0.470 (0.113; 0.828) | 0.605 | 0.014 |
|  | Self-Efficacy | 21 | -0.040 (-0.734; 0.654) | -0.036 | 0.903 |
| Trait food craving | PSS Total | 20 | 1.248 (0.067; 2.429) | 0.504 | 0.040 |
|  | Helplessness | 20 | 1.712 (0.448; 2.975) | 0.528 | 0.012 |
|  | Self-Efficacy | 20 | 0.492 (-1.988; 2.972) | 0.105 | 0.675 |
| **Normal weight** | | | | | |
| PFS Total | PSS Total | 46 | 0.004 (-0.032; 0.040) | 0.032 | 0.829 |
|  | Helplessness | 46 | 0.063 (-0.001; 0.128) | 0.338 | 0.055 |
|  | Self-Efficacy | 46 | 0.077 (-0.005; 0.158) | 0.314 | 0.066 |
| PFS Availability | PSS Total | 46 | 0.020 (-0.019; 0.058) | 0.145 | 0.314 |
|  | Helplessness | 46 | 0.068 (-0.003; 0.140) | 0.327 | 0.061 |
|  | Self-Efficacy | 46 | 0.047 (-0.044; 0.138) | 0.172 | 0.304 |
| PFS Present | PSS Total | 47 | 0.017 (-0.038; 0.072) | 0.097 | 0.530 |
|  | Helplessness | 47 | 0.087 (-0.016; 0.189) | 0.313 | 0.095 |
|  | Self-Efficacy | 47 | 0.076 (-0.053; 0.204) | 0.212 | 0.240 |
| PFS Taste | PSS Total | 47 | -0.023 (-0.064; 0.017) | -0.161 | 0.245 |
|  | Helplessness | 47 | 0.033 (-0.041; 0.107) | 0.148 | 0.366 |
|  | Self-Efficacy | 47 | 0.100 (0.007; 0.193) | 0.341 | 0.036 |
| Uncontrolled eating | PSS Total | 47 | 0.078 (-0.060; 0.215) | 0.171 | 0.262 |
|  | Helplessness | 47 | 0.229 (-0.030; 0.487) | 0.324 | 0.081 |
|  | Self-Efficacy | 47 | 0.125 (-0.199; 0.450) | 0.137 | 0.440 |
| Trait food craving | PSS Total | 46 | 0.177 (-0.483; 0.836) | 0.075 | 0.592 |
|  | Helplessness | 46 | 0.756 (-0.599; 2.112) | 0.202 | 0.266 |
|  | Self-Efficacy | 46 | 0.542 (-1.066; 2.149) | 0.116 | 0.499 |

Only coefficients for the focal PSS predictors from the final models including current dieting are shown. PSS Total was examined in separate models; Helplessness and Self-Efficacy were entered simultaneously in subscale models. Models in the primary analysis sample were adjusted for age, sex, BMI, current hunger, and current dieting. BMI-defined subgroup models were adjusted for age, sex, current hunger, and current dieting; BMI was omitted because the subgroups were defined by BMI. Current dieting was entered as a binary covariate. Abbreviations: B, unstandardized regression coefficient; β, standardized regression coefficient; CI, confidence interval; PFS, Power of Food Scale; PSS, Perceived Stress Scale.
